# Grid-Coded Retrieval-Augmented Generation with Hybrid Retrieval for Minute-Level Aircraft Trajectory Prediction

**DOI:** 10.3390/s26216821

**Published:** 2026-10-28

**Authors:** Yicen Liu, Fukuan Tang, Haibo Zhang, Shize Guo

**Affiliations:** 1 National Key Laboratory of Blind Signal Processing, Chengdu 611731, China; 2 School of Cyberspace Security, Beijing University of Posts and Telecommunications, Beijing 100876, China

**Keywords:** aircraft trajectory prediction, retrieval-augmented generation, grid discretisation, Markov chain, ADS-B, tactical conflict detection, air traffic management

## Abstract

**Highlights:**

**What are the main findings?**

- Minute-level tactical trajectory prediction is reformulated as next-grid classification and assessed by Top-K hit rate under data-leakage-free settings, which shows that the deep learning baselines, namely, multilayer perceptron (MLP), long short-term memory (LSTM) and gated recurrent unit (GRU), fall below the trivial Persistence baseline on short-horizon hit rate and incur an order of magnitude larger position error.
- A grid-coded dual-path retrieval framework (RAG-Hybrid) that couples deterministic breadth-first search (BFS) grid lookup with spatially aware FAISS (Facebook AI Similarity Search) soft retrieval matches the fair global-Markov baseline on single-step Top-1 (0.7097) and raises multi-candidate completeness at the +1 min horizon (Top-5 0.9839 against 0.9355), while on the pooled five-step protocol the same baseline remains marginally ahead (0.7548 against 0.7452); point accuracy is highest for the constant-velocity motion model (Top-1 0.8065) and candidate-set completeness for the proposed framework (Top-5 0.9839 against 0.8065).

**What are the implications of the main findings?**

- Lookup-based, training-free prediction retains a structural advantage over parametric learning when tactical grid-transition samples are sparse, so the framework can be deployed to new airspace sectors without retraining as grid configurations change, within the two-dimensional, 4032-cell grid studied here.
- Knowledge-base-coverage-guaranteed multi-candidate output matches the 1–5 min conflict-detection window, in which a missed transition costs more than a redundant candidate, whereas broader retrieval is preferable for longer prediction horizons.

**Abstract:**

Ultra-short-term (1–5 min) tactical trajectory prediction underpins air traffic conflict detection, yet deep learning models such as long short-term memory (LSTM), gated recurrent unit (GRU) and multilayer perceptron (MLP) networks lack an aviation spatial inductive bias, fit sparse grid-transition samples poorly, and in the small-sample setting studied here incur position errors an order of magnitude larger than trivial baselines. Retrieval-augmented generation (RAG) is a natural remedy, replacing parametric fitting with non-parametric retrieval of historical flight priors and requiring no retraining. Existing RAG schemes nevertheless break down on gridded airspace: character-hash encoding of grid identifiers discards spatial semantics, so adjacent grids are treated as unrelated, and the transition prior of the current grid is often missed, while stochastic vector search offers no recall guarantee, so such a miss silently collapses the prediction to persistence. This paper therefore proposes RAG-Hybrid, a grid-coded dual-path framework in which a deterministic breadth-first search (BFS) lookup reconstructs the local transition subgraph of the current grid from the knowledge base, spatially aware FAISS (Facebook AI Similarity Search) retrieval draws on a training-free focal-weighted Gaussian grid vectorisation, and Laplace smoothing with global-Markov weighting calibrates the resulting transition matrix. The evaluation uses Automatic Dependent Surveillance–Broadcast (ADS-B) trajectories from 62 test flights over a 0.25° × 0.25° grid covering Central–South China, and reports 1 min to 5 min horizons. On this test set, the framework holds single-step Top-1 at 0.7097, identical to a leakage-free global-Markov baseline and within one standard error of the trivial persistence predictor (0.7581), while raising Top-3 and Top-5 at the +1 min horizon from 0.9355 to 0.9839. A constant-velocity dead-reckoning baseline attains the highest point accuracy at this horizon (Top-1 0.8065, MAE 4.90 km) but returns a single cell, so the framework exceeds it on Top-5 by 0.1774. The one component effect that this study resolves statistically is the deterministic lookup channel at the far end of the horizon: removing it cuts the pooled five-step Top-5 hit rate from 0.7452 to 0.6516 and the +5 min Top-5 hit rate from 0.5645 to 0.3710.

## 1. Introduction

With the sustained expansion of global economic and trade activity, the demand for air passenger and cargo transportation has risen in parallel across the entire aviation industrial chain. Increasing traffic density, growing heterogeneity of vehicle performance, from conventional transport-category aircraft to emerging urban air mobility vehicles, and the progressive opening of low-altitude airspace have jointly rendered the airspace operating environment increasingly complex, imposing a growing operational burden and severe control pressure on air traffic management (ATM) systems [1]. To address these practical difficulties, major aviation economies have launched dedicated technological innovation programmes aimed at high-efficiency next-generation ATM frameworks. The United States has developed the Next Generation Air Transportation System (NextGen) [2] to advance the intelligent upgrading of its national airspace infrastructure, while the European Union has implemented the Single European Sky ATM Research (SESAR) programme [3] to remove the institutional and technical barriers to collaborative airspace scheduling among member states.

Accurate flight trajectory prediction is a core enabling technology of these frameworks. It delivers real-time, reliable data support for critical control tasks including aircraft conflict detection and resolution [4], dynamic air traffic flow management [5], and flight delay prediction [6]. The quality of the predicted trajectory directly bounds the quality of every downstream decision, and therefore determines how much look-ahead time a controller can be granted.

Trajectory prediction is not a monolithic task. Its accuracy requirement, admissible latency, and appropriate evaluation metric all vary fundamentally with the look-ahead horizon. At the strategic and pre-tactical levels, air traffic flow management operates over horizons of tens of minutes to several hours, where the useful quantity is an aggregate demand profile and metre-level position error is tolerable. At the tactical level, conflict detection and separation assurance operate over a window of 0–10 min, and the decisive interval is 1–2 min; this is the window within which a controller must decide whether to issue a resolution advisory, and within which a false negative is operationally unacceptable.

The current literature is heavily skewed towards the former regime. The dominant majority of reported results are expressed as root mean square error (RMSE), mean absolute error (MAE), average displacement error (ADE), or final displacement error (FDE), evaluated at 10–60 min look-ahead. Position error expressed in metres is nevertheless a poor proxy for tactical utility. What a controller requires is not a single point estimate with an uncertainty radius, but an answer to an operational question, i.e., which airspace unit will the aircraft occupy next, and how many candidate units must be monitored to be confident? A model that reduces average position error from 4.9 NM to 3.4 NM over 10 min conveys little about whether its +1 min prediction can be trusted to trigger or withhold an intervention. Consequently, a systematic capability gap persists in the 0–10 min horizon, and more acutely, in the 0–2 min interval where the cost of a missed detection is highest. Existing prediction approaches do not satisfy the control demands of this critical time window.

Prior art for aircraft tactical trajectory prediction falls into four families, each with a structural bottleneck in accuracy, deployability or small-sample adaptability [1]. Rule-based and statistical methods, including persistence and constant-velocity dead reckoning, have minimal computational overhead [7]. First-order Markov models, however, suffer from context-blind averaging [8,9], and higher-order Markov models from explosive parameter growth under sparse observations [10,11,12]; the discounting and smoothing techniques developed for sparse frequency estimation [13] reduce the variance of such estimates but at the cost of a context-independent bias. Nonparametric similarity-retrieval methods match historical trajectories by prefix similarity, using GPU-accelerated nearest-neighbour indexing [14], meta-learned similarity metrics [15] or deep metric learning over historical scenarios [16]; their similarity metric relies on continuous spatial proximity, which is destroyed once trajectories are discretised into airspace grids. Deep learning is the dominant family and delivers strong predictive performance; its taxonomy is reviewed in [17], and its architectures are typically recurrent encoders [18,19] or attention mechanisms [20,21,22,23,24], with the statistical-learning-theoretic account of their sample requirements given in [25]; the 4-D trajectory-prediction literature is surveyed in [26]. These models nevertheless depend on large, labelled trajectory datasets and transfer poorly across sectors. Recent retrieval-augmented generation (RAG) methods integrate nonparametric retrieval with parametric models, generically [27], through scalable vector search [28], in time-series foundation models [29], in spatio-temporal prediction [30] and in supervised next-location prediction [31]. Existing RAG frameworks, however, do not control retrieval recall, discard the spatial semantics of discretised grids, and therefore yield high-variance transition probability estimates for grid-based trajectory prediction.

Across all four families of approaches, three recurring bottlenecks can be identified. (1) Coverage limitation. No existing mechanism ensures that the local transition prior corresponding to the current state can be obtained during inference. As a result, every method may degrade to the persistence lower bound under worst-case conditions. (2) Context conditioning under small-sample scenarios. Models either aggregate all flight records to build a context-free global transition matrix, or learn context-dependent patterns directly from data. Small sample sizes prevent models from completing such a learning process effectively. (3) Deployability constraint. Prediction accuracy is tightly coupled with the total number of airspace grids. Deep learning models require retraining of learned embeddings when grid configuration changes, while higher-order Markov models demand storage resources scaling at the order of *O*(*G^k^*^+1^). Neither type of method can be migrated from an experimental setup with 180 grids to nationwide airspace without system re-engineering.

This paper proposes a grid-coded retrieval-augmented framework named RAG-Hybrid to jointly tackle the three identified bottlenecks. The framework discretises 1 min-sampled ADS-B trajectories into grid sequences and unifies diverse prediction strategies via Markov power iteration, supporting rigorous quantitative assessment of short-term prediction accuracy using stepwise Top-K hit rates. RAG-Hybrid integrates deterministic matching with stochastic retrieval. Its breadth-first deterministic retrieval branch b, built upon grid outbound statistics, reconstructs the local transition subgraph that is recorded in the knowledge base, and the corresponding outputs are fused with spatially aware soft retrieval results obtained from FAISS. More specifically, three core design components of RAG-Hybrid directly target the aforementioned bottlenecks. Training-free grid-ID-aware spatial vectorisation adopts a Gaussian kernel over grid coordinates with focal weighting. It recovers physical spatial semantics within the retrieval space without learned parameters, and ranks similarity strictly according to geographic proximity, which addresses the problem of context conditioning under small-sample scenarios. The deterministic breadth-first branch makes the recall of that subgraph independent of the similarity metric, which resolves the coverage limitation problem at the level of the knowledge base, though not for transitions the training flights never exhibited (Section 4.2). Laplace smoothing combined with global-Markov weighted fusion mitigates the variance of sparse transition counts while retaining local specificity. Since the estimator relies on lookup over observed local transition subgraphs instead of parametric function approximation, its sample complexity does not scale with *G*. Unlike deep models that estimate conditional distributions over *G* candidates with G-proportional parameters, direct counting only requires *O*(1/*ξ*^2^) samples per outgoing edge and stays independent of the global grid count. This structural property delivers superior performance under small-sample settings and addresses the problem of deployability constraints.

This paper comprehensively validates the performance of the proposed method on real-world Automatic Dependent Surveillance–Broadcast (ADS-B) data, with ten mainstream baseline models for comparison, including classical statistical models, nonparametric models, and state-of-the-art deep learning models. The core research contributions of this work are summarised as follows.

(1) This work innovatively reformulates the minute-level tactical aircraft trajectory prediction task. Traditional methods typically adopt metre-level regression paradigms that focus on coordinate fitting errors. In contrast, we discretise the continuous airspace and convert the original trajectory regression problem into a next-grid classification task. The aircraft flight evolution is further modelled as a spatially constrained inhomogeneous semi-Markov process, which bridges the long-standing mismatch between conventional regression-based evaluation metrics and practical tactical air traffic control demands. Moreover, a unified Markov power-iteration inference framework and Top-K hit-rate evaluation protocol are established, enabling fair and consistent quantitative comparisons among statistical, nonparametric, deep learning-based, and retrieval-based prediction methods.
(2) This work addresses the inherent limitations of existing deep learning models in ultra-short-term, small-sample tactical prediction scenarios, including the lack of aviation-specific spatial inductive bias, poor fitting performance on sparse grid transition samples, and large positional prediction errors. To tackle these issues, we propose a training-free hybrid retrieval prediction paradigm. The designed grid-ID-aware spatial vectorisation mechanism effectively preserves inherent airspace spatial semantics. When ideal retrieval fails, the method can smoothly switch to neighbouring spatial grid searching instead of producing invalid predictions. Free from training dependency, the proposed approach requires no retraining when airspace grid partitions are adjusted, significantly improving deployment flexibility in real-world air traffic scenarios.
(3) A dual-path hybrid retrieval framework is proposed to address the retrieval-omission failure of single-path RAG prediction. By combining a deterministic BFS lookup over recorded grid out-edges with spatially aware soft retrieval, the framework removes the dependence of recall on the similarity metric, which is the mechanism that makes conventional retrieval-only variants degenerate into persistence prediction. The two channels are complementary rather than jointly necessary: the deterministic channel fixes the support of the local transition row, while the soft channel supplies a supplementary prior from analogous historical flights.
(4) The framework is characterised as an operating-point choice between single-step precision and multi-candidate completeness rather than as a uniform improvement. On the leakage-free test set, it matches the global-Markov baseline exactly on single-step Top-1 (0.7097 for both, and identical first-ranked grids for all 62 test flights), raises Top-3 and Top-5 at the +1 min horizon from 0.9355 to 0.9839, and does not improve on that baseline in the pooled five-step protocol, where the baseline is marginally ahead on Top-5 (0.7548 against 0.7452). The reported gains over the parametric baselines are larger and are the ones this study establishes: at the +1 min horizon the deep baselines reach Top-1 of 0.5323 (GRU), 0.4032 (MLP) and 0.3548 (LSTM) against 0.7097, and their mean absolute position error is 84.28–118.54 km against 7.35 km. Inference is lightweight, and its deployment cost does not scale with the number of grids, which is what makes the framework applicable where grid configurations change.

The remainder of this paper is structured as follows. Section 2 reviews the existing literature on data-driven trajectory prediction, grid-discretised Markov modelling, trajectory representation learning, and retrieval-augmented generation, and systematically identifies the core research gaps in current studies. Section 3 formulates the minute-level grid trajectory prediction problem and elaborates the grid coding strategy, Markov power-iteration rollout mechanism, and Top-K evaluation criterion. Section 4 details the proposed framework, including grid-ID-aware spatial vectorisation, the baseline retrieval model, and the dual-path RAG-Hybrid architecture, as well as the Laplace smoothing and global weighted fusion strategies. Section 5 conducts extensive experiments on real-world ADS-B datasets to evaluate the proposed methods from prediction accuracy, Pareto optimality, computational complexity and deployability perspectives and further analyses the application scenarios of the proposed framework. Section 6 concludes this paper and prospects the practical engineering deployment of the proposed approach.

## 2. Related Work

The proposed method lies at the intersection of four research lines: data-driven aircraft trajectory prediction, grid discretisation and Markovian trajectory modelling, trajectory representation learning and grid vectorisation, and retrieval-augmented generation. This section reviews state-of-the-art studies in each domain and highlights the unresolved research gaps addressed in this paper.

### 2.1. Data-Driven Aircraft Trajectory Prediction

With the widespread deployment of Automatic Dependent Surveillance–Broadcast (ADS-B) equipment, data-driven methods have become the mainstream paradigm for aircraft trajectory prediction. A recent comprehensive survey [17] systematically summarises deep learning-based trajectory prediction studies and categorises existing models into five typical architectures, including recurrent neural networks, attention-based models, generative models, graph neural networks, and hybrid frameworks. Current research predominantly adopts RMSE, MAE, ADE, and FDE as standard evaluation metrics.

Despite their promising performance in capturing medium- and longer-horizon trajectory trends, existing data-driven methods exhibit three inherent limitations for tactical air traffic management scenarios. First, most deep learning models operate as black-box systems without explicit airspace semantic interpretability, which hinders their practical deployment for safety-critical aviation tasks. Second, these models rely heavily on large-scale labelled trajectory datasets, resulting in limited generalisation ability across unseen airspace sectors and requiring full retraining for environmental adaptation. Third, existing evaluations mainly focus on meter-level positional errors over 10–60 min prediction horizons, while classification-based hit-rate metrics for minute-level short-term prediction, critical for tactical conflict detection, are rarely adopted.

Tactical air traffic decision-making typically requires a 1–2 min look-ahead horizon with stringent accuracy and confidence requirements, where missed conflicts incur far higher operational risks than redundant advisories. However, few existing studies specifically target this tactical-level prediction demand or guarantee performance improvement over the persistence baseline. This paper focuses on minute-scale short-term trajectory prediction, adopting a 1 min ADS-B sampling interval to fit tactical decision cycles and establishing performance guarantees beyond the persistence lower bound.

Representative data-driven trajectory prediction methods have made progressive improvements in aviation prediction accuracy yet still suffer from structural defects. Shi et al. [32] proposed a constrained LSTM (cLSTM) that integrates physical navigation constraints into model optimisation and adopts Markov-based baselines for 4D trajectory prediction. Nevertheless, the regression-based paradigm requires elaborate feature engineering and cannot be directly generalised to new airspace environments without retraining. Tran et al. [33] designed an encoder–decoder structure to model aircraft tactical intent, reducing positional error to meet EUROCONTROL standards, yet the dependence on labelled intent information limits its applicability to raw, unprocessed ADS-B data. Choi et al. [34] developed a hybrid GMM-LSTM prediction framework embedded with a residual interacting multiple model, achieving superior terminal-area prediction accuracy at the cost of extremely complex system architecture and limited cross-scenario transferability. Recent generative prediction methods have explored probabilistic trajectory modelling but still follow continuous regression paradigms and fail to match tactical evaluation requirements.

### 2.2. Grid Discretisation and Markovian Trajectory Modelling

Grid discretisation combined with Markovian transition modelling is a classical interpretable trajectory prediction paradigm that balances computational efficiency and physical rationality. Ayhan and Samet [35] constructed a three-dimensional grid-based airspace representation and adopted a hidden Markov model (HMM) integrated with meteorological information to decode optimal flight trajectories via the Viterbi algorithm. Faulkner and McFadyen [36] modelled dynamic sector traffic occupancy based on discrete-time Markov chains and estimated state transition probabilities using the Baum–Welch algorithm to support dynamic airspace resource allocation.

Pan [37] further advanced grid-based Markov prediction by constructing multi-step transition probability matrices for up-to-20 min trajectory forecasting, achieving significant error reduction compared with conventional baselines. Although this method validates the effectiveness of the grid–Markov paradigm, its global statistical transition matrix ignores trajectory-specific contextual information, where all flights within the same grid share identical transition distributions regardless of their historical arrival paths. Moreover, data sparsity in real-world ADS-B observations frequently leads to insufficient grid transition samples, degrading model outputs to self-loop persistence predictions.

In terms of spatial discretisation schemes, Ostroumov [38] introduced Uber H3 hexagonal grid indexing for ADS-B trajectory encoding, which conforms to performance-based navigation uncertainty characteristics and provides hierarchical spatial precision. Extended research [39] further applied H3 grids to airspace partitioning and trajectory classification. However, hexagonal grid structures are incompatible with prevailing latitude–longitude rectangular airspace management specifications, introducing high practical deployment costs. In addition, existing H3-based studies only focus on spatial representation rather than predictive modelling.

Overall, grid-Markov methods possess excellent interpretability, yet they suffer from context-independent global statistical modelling and sparse data degradation issues. Most importantly, the existing literature fails to integrate retrieval-augmented mechanisms into grid-based prediction frameworks to construct context-aware local transition distributions, which constitutes a core research gap addressed in this paper.

### 2.3. Trajectory Representation Learning and Grid Vectorisation

Trajectory representation learning maps raw flight sequences into low-dimensional embedding vectors to support downstream prediction and analysis tasks. Recent studies mainly focus on multi-modal representation fusion for grid and road network scenarios. TIGR [40] and GREEN [41] adopt contrastive learning and masked reconstruction to fuse grid and road network features, achieving superior performance in trajectory similarity matching and arrival time prediction. NextLocLLM [42] leverages large language models to encode continuous coordinate semantics for enhanced cross-domain generalisation.

In airspace grid representation research, Deng et al. [43] proposed 3SGNN, which constructs grid adjacency graphs and employs graph neural networks to learn spatially aware grid embeddings, yielding substantial performance gains in sub-trajectory retrieval. This paper verifies the necessity of maintaining spatial topological adjacency in embedding space. However, GNN-based embedding learning relies on complete labelled datasets and requires retraining for unseen grids, limiting its adaptability to dynamic airspace scenarios and short-term stepwise prediction tasks. In aviation traffic forecasting, Cai et al. [44] proposed TAaDGCN, which integrates graph convolution and spatial embedding to capture sector-level traffic patterns. Nevertheless, its fixed sector-dependent graph structure cannot be adapted to fine-grained grid-based airspace representation.

Existing representation learning methods fully demonstrate the significance of spatial semantics for trajectory modelling. However, most data-driven embedding schemes require massive training resources and are tailored for urban traffic or fixed-sector aviation scenarios. No current study develops training-free geometric grid vectorisation tailored for discrete airspace prediction, which motivates the grid-ID-aware Gaussian kernel vectorisation design in this paper.

### 2.4. Retrieval-Augmented Generation

Retrieval-augmented generation (RAG) integrates nonparametric vector retrieval with a parametric generative model, so that external knowledge can be injected without full retraining [27]. The FAISS vector search engine adopted in this paper belongs to the same line of work and supports billion-scale high-speed vector retrieval [28]. Gao et al. [45] systematically classified RAG development into naive, advanced and modular paradigms and showed that embedding quality and retrieval accuracy dominate overall model performance. That finding bears directly on the present problem: because the retrieval space of a grid-coded trajectory is determined entirely by the embedding, an embedding that does not preserve spatial adjacency necessarily limits retrieval quality.

Introducing RAG into trajectory prediction enables flexible integration of historical flight priors, which is particularly suitable for small-sample tactical aviation scenarios. However, existing trajectory RAG methods adopt generic string-based grid encoding, where vector similarity depends purely on character matching rather than physical airspace spatial proximity. Spatially adjacent grids with similar geographic locations may present irrelevant embedding distributions, leading to invalid retrieval results and missing critical local transition priors. Such spatial semantic blindness severely degrades the stability and accuracy of tactical one-step prediction, yet this key defect has not been resolved in existing studies.

### 2.5. Research Gaps and Motivations

Based on the above literature review, as shown in Table 1, no method family other than the proposed RAG-Hybrid satisfies all six criteria simultaneously; every previous family fails on at least two. This paper summarises four core research gaps that motivate the proposed framework. First, existing data-driven trajectory prediction methods focus on medium- and long-term forecasting with meter-level positional error metrics, lacking targeted designs for minute-level tactical prediction and classification-based hit-rate evaluation paradigms. No existing method guarantees stable performance beyond the persistence baseline under strict tactical decision-making constraints. Second, conventional Markov-based grid prediction methods rely on global static transition statistics without trajectory-level contextual conditioning. Sparse observation data frequently degrade model outputs to persistence self-loops, and no existing framework provides deterministic coverage guarantees for local grid transition modelling. Third, current retrieval-augmented trajectory models are decoupled from airspace spatial semantics. Data-driven spatial embedding methods require retraining and fixed topological structures, while generic RAG vectorisation cannot reflect geographic grid adjacency, resulting in invalid prior retrieval for tactical prediction. Fourth, deep learning models suffer from severe sample complexity mismatch for grid-based aviation prediction. Their parameter scale grows with airspace grid numbers, leading to insufficient sample-to-parameter ratios and performance degradation under small-sample tactical scenarios. In contrast, lookup-table-based statistical estimation adopted in this paper achieves grid-agnostic sample complexity, providing inherent structural advantages for sparse trajectory prediction.

Targeting the above gaps, this paper proposes a semantics-aware, coverage-guaranteed hybrid retrieval framework for minute-scale tactical trajectory prediction. The developed training-free spatial vectorisation embeds physical airspace semantics into retrieval processes, while the dual-path retrieval structure ensures complete local transition coverage and contextual modelling. The proposed method establishes a lightweight, stable, and interpretable tactical prediction paradigm that fills the technical gap between existing trajectory prediction research and practical ATM tactical decision requirements.

Contribution relative to the reviewed literature. The four lines above each solve part of this problem, and the contribution of this work is best stated as the four increments that none of them supplies, together with an explicit account of what it does not supply. Relative to data-driven trajectory prediction (Section 2.1), which reports metre-level displacement error over horizons of tens of minutes and requires retraining for each airspace, this work replaces the regression target with a next-grid classification assessed by a hit rate that maps onto the number of airspace units a controller must monitor, and it removes the retraining requirement. Relative to grid-Markov modelling (Section 2.2), which applies one global transition row to every flight passing through a grid, this work conditions the row on the observed arrival context, so that the same grid yields a different successor distribution under a different recent history. Relative to trajectory representation learning (Section 2.3), which learns grid embeddings from labelled data and fixes a graph topology, this work derives the embedding analytically from grid adjacency and therefore needs neither training nor a fixed topology. Relative to retrieval-augmented generation (Section 2.4), which relies on a single stochastic search channel whose recall is uncontrolled, this work adds a deterministic channel over recorded grid out-edges and bounds explicitly what that channel covers. The scientific claim that follows is deliberately narrower than that of earlier versions of this work: a training-free lookup estimator, once its recall is made independent of the similarity metric and its spatial semantics are encoded geometrically, reproduces the single-step accuracy of a leakage-free global-Markov baseline exactly and does not improve on its pooled multi-candidate completeness; what it does establish is a large and statistically resolved advantage over parametric learners in the small-sample regime, concentrated at the far end of the ultra-short-term horizon, obtained without training and without a deployment cost that scales with the number of grids.

## 3. Problem Description and Formal Modelling of Minute-Scale Tactical Trajectory Prediction for Aircraft

### 3.1. Air-Domain Temporal Modelling Based on Semi-Markov Process

#### 3.1.1. Minute-Scale Sampling Paradigm for ADS-B Trajectories

Automatic Dependent Surveillance-Broadcast (ADS-B) for aircraft continuously outputs four-dimensional flight state information, including longitude-latitude coordinates, altitude, ground speed, and heading angle, which fully characterise the short-term manoeuvre evolution features of aircraft. Targeting the 1–5 min tactical decision-making window for air traffic control, this paper adopts a fixed-interval sampling mechanism with a 1-min time step to discretise continuous time-series trajectories into structured observation sequences:

*T* = {*x_t_* = (*lon_t_*,*lat_t_*,*alt_t_*,*v_t_*,*ψ_t_*) ∈ *R*^5^ | *t* ∈ *Z*^+^} (1)

where *v_t_* and *ψ_t_* denote the instantaneous ground speed and heading angle, respectively. The discrete sampling paradigm strictly aligns with the tactical operational timescale, such that the predicted state at time step *t* + *n* corresponds to the aircraft spatial distribution after *n* minutes. This standardised temporal discretisation establishes a unified evaluation benchmark for minute-level tactical prediction.

#### 3.1.2. Airspace Gridding and Discrete State Space Definition

**Figure 1:**
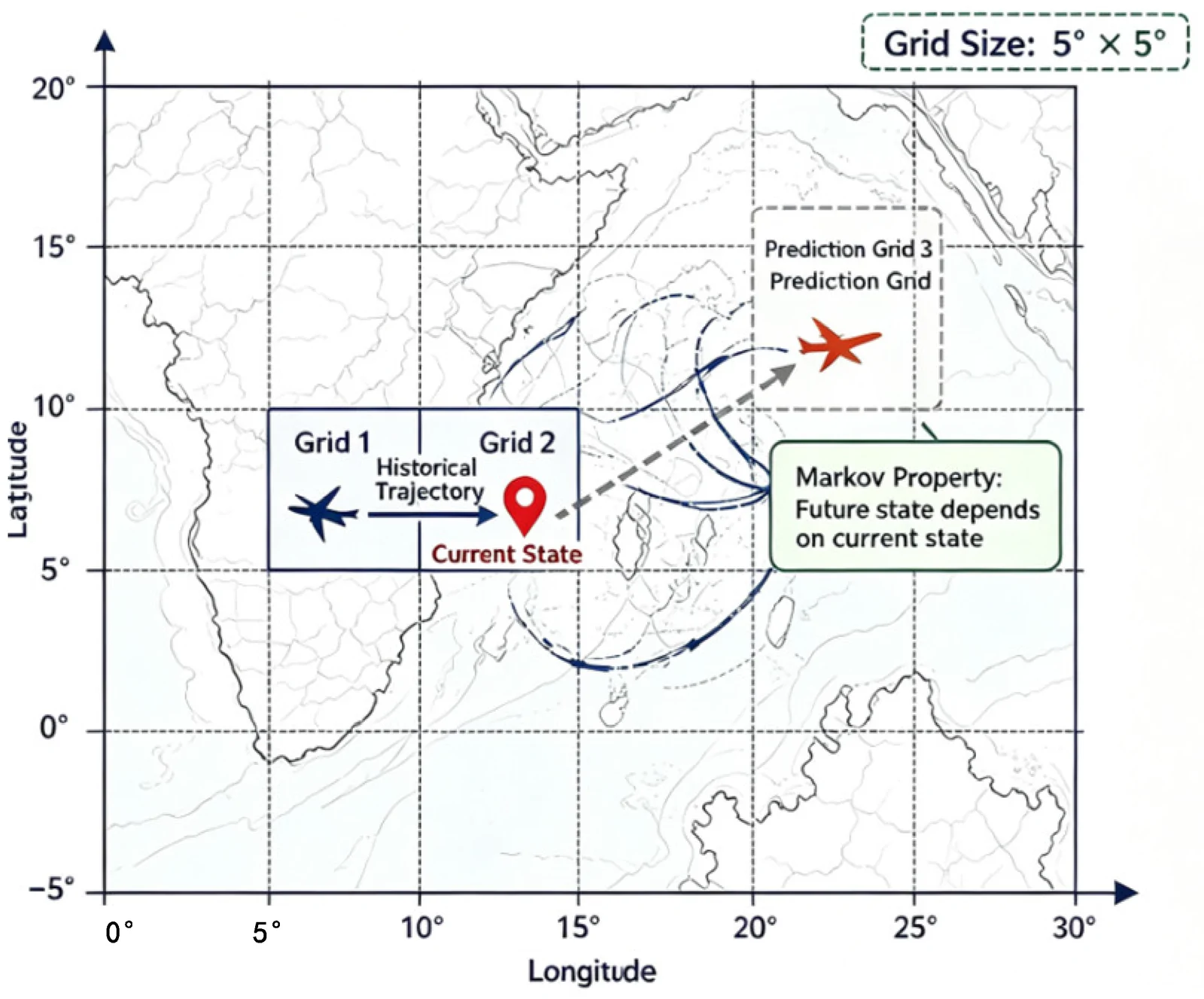
Illustration of Markovian grid-based flight trajectory prediction.

This paper addresses minute-scale tactical aircraft trajectory prediction by abstracting continuous aircraft spatial motion into discrete state transitions over gridded airspace. This transformation recasts conventional continuous coordinate regression into a discrete-state stochastic classification task, which fits the operational logic of grid-based tactical conflict assessment. Figure 1 visualises the Markovian trajectory prediction paradigm developed for this problem. For an aircraft travelling from grid 1 to grid 2, the model predicts the aircraft’s next accessible grid (grid 3) using only the current grid occupancy. This modelling formulation satisfies the Markov property: future spatial states are conditionally independent of historical trajectory information given the present state.

The state vector of Equation (1) contains altitude, but the index mapping of Equation (2) is two-dimensional, so the predicted state is a horizontal cell and vertical motion is projected onto the horizontal plane. Two consequences are stated here rather than left implicit. First, a climb or descent is recorded as a horizontal transition whenever it is accompanied by horizontal displacement, so part of the residual error of any method in this paper is vertical motion seen through a planar representation; for the cruise-dominated traffic studied here this is a second-order effect, and the disagreement that remains between methods is confined to sparsely sampled boundary cells. Second, the framework as formulated cannot support a vertically resolved separation assessment, and the operational claims made here are restricted accordingly.

The airspace can be partitioned into *M* × *N* uniform grids, yielding a finite discrete state space *G* = {*g*_1_, *g*_2_, …, *g*_|*G*|_}. For any geodetic coordinate (*lon*, *lat*), the grid indexing mapping is defined as

(2) $$\left\{\begin{array}{l} i_{\mathrm{lon}}=\left\lfloor \frac{\mathrm{lon}-{\mathrm{lon}}_{\min}}{\Delta_{\mathrm{lon}}}\right\rfloor ,\;\Delta_{\mathrm{lon}}=\frac{{\mathrm{lon}}_{\max}-{\mathrm{lon}}_{\min}}{M}\\ j_{\mathrm{lat}}=\left\lfloor \frac{\mathrm{lat}-{\mathrm{lat}}_{\min}}{\Delta_{\mathrm{lat}}}\right\rfloor ,\;\Delta_{\mathrm{lat}}=\frac{{\mathrm{lat}}_{\max}-{\mathrm{lat}}_{\min}}{N} \end{array}\right.$$

Each grid is assigned a unique spatial identifier *g* = *G*(*i_lon_*, *j_lat_*). Consequently, a continuous flight trajectory is converted into a discrete state sequence:

*S* = (*g*_1_, *g*_2_, …, *g_L_*), *g_t_* = *G*(*x_t_*) ∈ *G*, ∀*t* ∈ *Z*^+^ (3)

This transformation converts the conventional continuous coordinate regression problem into a discrete state stochastic classification task, which is more consistent with the tactical grid-based conflict assessment logic.

Choice and sensitivity of the grid resolution. The resolution is not a free hyperparameter; it is fixed by three requirements that follow from the tactical task, and it was checked against the observed displacement statistics rather than tuned on the evaluation set. (i) Displacement scale. One minute of cruise flight at 400–900 km h^−1^ corresponds to 6.7–15.0 km of ground track. On the World Geodetic System 1984 (WGS-84) ellipsoid, a 0.25° cell edge spans 27.8 km in latitude and 26.5 km in longitude at 18° N, falling to 22.5 km at 36° N. The cell edge is therefore 1.5 to 4 times the one-minute displacement, which is what makes the successor distribution non-degenerate: at the measured self-loop ratio of 0.5965, roughly three steps in five stay inside the current cell and the remaining two distribute over distinct successors. A coarser cell would raise the self-loop ratio, which increases monotonically with the ratio of cell edge to one-minute displacement, and would drive the task towards persistence; a finer cell would divide the per-grid transition counts that the lookup estimator depends on, and those counts are already thin, averaging 4.0 outgoing observations per grid in the knowledge base (9913 edges over 2484 grids). The chosen resolution is thus the coarsest one that still resolves the one-minute displacement and the finest one that the available sample count supports. (ii) Administrative compatibility. 0.25° is exactly 15 arc-minutes, an integer sub-multiple of the 1° × 1° cell used in prevailing latitude–longitude airspace and meteorological products, so the grid nests exactly inside operational reference cells without splitting them. (iii) Sensitivity. Because every reported quantity is a ratio over a finite test set, resolution enters the results only through the self-loop ratio and the mean number of distinct successors per grid, and the argument given here is a design justification supported by reported statistics, not a substitute for that sweep.

#### 3.1.3. Stochastic Evolution Modelling via Semi-Markov Process

Standard homogeneous Markov chains assume constant state residence duration and stationary transition kernels, which fail to characterise the inhomogeneous temporal residence, manoeuvre-induced time-varying transitions, and sparse topological jumps of real-world aircraft trajectories. To overcome these limitations, this paper formulates aircraft trajectory evolution as a spatially constrained inhomogeneous Semi-Markov Process (SMP) with two core stochastic components: state residence time distribution and spatial transition kernel.

**Definition** **1** (Grid State Residence Time)**.** *Given a discrete airspace state g* ∈ *G, the state residence time τ_g_ is defined as a nonnegative integer-valued random variable obeying a state-dependent probability mass function:*

(4) $$F_{g}(\tau )=\mathrm{Pr}(\tau_{g}=\tau |g)=\frac{\tau_{\lambda }^{g}e^{-\lambda_{g}}}{\tau !},\tau \in N^{*}$$

*where λ_g_ denotes the state-specific dwell intensity parameter determined by historical flight statistics within grid g. This Poisson-distributed residence modelling relaxes the fixed-step constraint of Markov chains and precisely characterises variable flight durations caused by cruising, climbing, turning, and speed adjustment*.

The reason why the residence time is modelled as Poisson can be expressed as follows. Three requirements fix this choice. First, the modelled quantity is a count: the number of consecutive one-minute samples during which the aircraft occupies grid *g*, a non-negative integer. Second, only its first moment is identifiable at the available sample size, because the knowledge base holds 9913 transition edges over 2484 grids, so a grid supports on average 4.0 outgoing observations and a two-parameter dwell law would be estimated from a handful of dwells. Third, the law must admit a closed form inside the discrete rollout of Equation (11). The Poisson law satisfies all three: it is the maximum-entropy distribution on the non-negative integers for a given mean, it is the small-per-step leaving-probability limit of the binomial count of stay-or-leave decisions accumulated over a sojourn, and it is closed under the sampling operation the estimator performs. The principal alternatives are less suitable here. A geometric dwell law imposes a constant leaving hazard, hence a memoryless sojourn, and the manoeuvre structure of the data is not memoryless in this sense, because climb, cruise, turn and speed-adjustment phases produce dwells that are neither memoryless nor single-parameter; it is for that reason not adopted as the model-level definition. It is nevertheless the law that the one-minute estimator instantiates, since a transition matrix estimated by counting one-minute transitions can carry a dwell only through the geometric continuation probability of its diagonal. A negative binomial law accommodates the over-dispersion induced by mixing over manoeuvre phases but needs a dispersion parameter that the per-grid sample cannot support. Continuous laws such as Weibull or log-normal describe dwell in minutes but have no closed form under one-minute discretisation. Equation (4) is written in its normalised form so that *λ_g_* is the mean residence time of grid *g*, and it is estimated per grid from the mean observed dwell in the knowledge base, which is the quantity recovered from the diagonal of Equation (9). What matters for the implementation is that the dwell law reaches the recursion only through this mean: the one-minute transition matrix is the sampling-grid image of the dwell law, so the reported hit rates are fixed by the continuation probability that its diagonal records, and the shape of *F_g_*(·) beyond that first moment does not enter them.

**Definition** **2** (Context-Conditional Spatial Transition Kernel)**.** *Different from unconditional global transition statistics, this paper defines a history-dependent state transition kernel conditioned on observational trajectory context H*_1:*t*_*:*

(5) $$K(g\to g^{\prime })≜P(g_{t+1}=g^{\prime }|g_{t}=g,H_{1:t})=\frac{p(g^{\prime },H_{1:t})}{p(g,H_{1:t})}$$

The transition probability is governed by both spatial topological adjacency and temporal flight patterns, satisfying the Markovian property of conditional independence of future states from remote history. Accordingly, the joint stochastic evolution of aircraft trajectory can be formally expressed as

(6) $$P\left(g_{t+\tau }=g^{\prime }|g_{t}=g\right)=\sum_{\tau =1}^{\tau_{\max}}F_{g}\left(\tau \right)\cdot K^{\tau }\left(g\to g^{\prime }\right)\cdot I\left(\tau \leq \tau_{\mathrm{residual}}\right)\;$$

This formulation integrates temporal residence stochasticity and spatial transition uncertainty, constructing a complete stochastic description for minute-level tactical flight evolution.

Focusing on the operational requirements of tactical conflict detection, this paper defines the short-term grid prediction task as a conditional probabilistic inference problem. Given the historical state sequence *H*_1:*t*_ = (*g*_1_, *g*_2_, …, *g_t_*), the model infers the future multi-step grid state distribution:

(7) $$F:H_{1:t}\mapsto \left\{{\hat{g}}_{t+1},{\hat{g}}_{t+2},\ldots ,{\hat{g}}_{t+n}\right\}$$

(8) $${\hat{g}}_{t+k}=\arg\underset{g\in G}{\max}P\left(g_{t+k}=g|H_{1:t}\right)$$

where *n* ∈ {1, 2, …, 5} denotes the tactical prediction horizon. The 1-step (1-min) prediction is taken as the primary evaluation metric, which matches the ultra-short operational lead time of tactical air traffic decision-making.

### 3.2. Unified Probabilistic Inference Framework via Markov Power Iteration

To ensure fair quantitative comparison and unified theoretical interpretation of different prediction paradigms, this paper establishes a generalised Markov power iteration inference architecture.

#### 3.2.1. First-Order Spatial Transition Matrix

Define the global stochastic transition matrix *T* ∈ *R*^|*G*|×|*G*|^, whose entries represent the one-step conditional transition probability between grid states:

(9) $$T_{g,g^{\prime }}=P\left(g^{\prime }|g\right),\;\;\;s.t.\;T_{g,g^{\prime }}\geq 0,\;\sum_{g^{\prime }\in G}T_{g,g^{\prime }}=1,\;∥T{∥}_{2}\leq \sqrt{\left|G\right|}$$

#### 3.2.2. Multi-Step Probability Power Iteration

Given the current grid state *g_t_*, the initial spatial probability distribution is formulated as a Dirac delta sparse vector:

(10) $$\pi_{0}=e_{g_{t}}\in \{0,1{\}}^{\left|G\right|},\pi_{0}(g)=\delta (g,g_{t})=\left\{\begin{array}{cc} 1, & g=g_{t}\\ 0, & g\neq g_{t} \end{array}\right.$$

where *δ*(·,·) denotes the Kronecker delta function. Based on the homogeneous Markov propagation rule, the *n*-step future probability distribution is derived via matrix power iteration:

*π_n_* = *π*_0_*T^n^* = *π_n_*_−1_*T*,*π_n_* = *E*[*π_n_*∣*π_n_*_−1_] (11)

To further regularise sparse probability estimation and enhance numerical stability, this paper introduces a spatial Laplace smoothing constraint into iterative propagation:

(12) $${\overset{\sim }{\pi }}_{n}=(1-\alpha )\pi_{n}+\alpha G_{\sigma }\pi_{n},G_{\sigma }={-1}_{D}^{\sigma }W_{\sigma },[W_{\sigma }{]}_{g,g^{\prime }}=\exp\left(-\frac{d(g,g^{\prime }{)}^{2}}{2\sigma^{2}}\right)$$

where *G_σ_* denotes the neighbourhood smoothing operator defined by Equation (12), applied to the row of the transition matrix associated with the current grid, so that the smoothed mass at grid *g* is a weighted average of the unsmoothed mass over *g* and its immediate neighbours; *d*(·,·) is the distance between grid centroids measured in cell units, the neighbourhood is the 8-connected Chebyshev ball of radius one, *W_σ_* is the corresponding unnormalised kernel matrix and *D_σ_* is its row-sum normaliser. Here *β* is the smoothing intensity, where *β* ≡ *α*; *β* = 0 removes the term and *β* = 1 replaces each row by the neighbourhood average.

#### 3.2.3. Top-K Tactical Evaluation Metric

To quantitatively evaluate tactical prediction accuracy and distribution completeness, this paper defines the Top-K hit rate metric over the entire test dataset, *D*:

(13) $${\mathrm{Acc}@K}_{n}=\frac{1}{\left|D\right|}\sum_{S\in D}1\left({\mathrm{true}}_{g}^{t+n}\in \mathrm{TopK}\left(\pi_{n}\right)\right),\;\mathrm{TopK}\left(\pi_{n}\right)={\mathrm{argmax}}_{g\in G}\pi_{n}\left(g\right)$$

where 1(·) is the indicator function. We adopt *Acc*@1 for precise deterministic prediction evaluation and *Acc*@5 for probabilistic distribution integrity evaluation, forming a comprehensive tactical prediction assessment system.

Two further definitions are needed to read the reported tables. The confidence metric *P*(true) is the model’s own probability estimate for the event that actually occurs: for a prediction sample whose anchor is *g_t_* and whose realised next cell is g^*^, *P*(true) = *π_n_*(g^*^), and the tabulated value is its mean over the evaluated samples. It is a calibration metric and not an accuracy metric, and it can only be read alongside a hit rate, since a model that concentrates all mass on one cell attains *P*(true) = 1 when correct and *P*(true) = 0 when wrong. The distance metrics are defined between grid centroids: for a sample with realised cell g^*^ and predicted cell $\hat{g}$, $\mathrm{MAE}=\frac{1}{N}\sum_{i}d\left(c\left({\hat{g}}_{i}\right),c\left({g^{*}}_{i}\right)\right)$ and $\mathrm{RMSE}=\sqrt{\frac{1}{N}\sum_{i}{d\left(c\left({\hat{g}}_{i}\right),c\left({g^{*}}_{i}\right)\right)}^{2}}$, where *c*(·) is the centroid of a cell computed on the World Geodetic System 1984 (WGS-84) ellipsoid and *d*(·,·) is geodesic distance. Grid centres are used, and not the aircraft’s instantaneous position, so the error of any grid predictor is quantised in multiples of the cell spacing. That quantisation is what makes the reported error magnitudes interpretable, and it is why the distance metrics and the hit-rate metrics are reported together rather than one replacing the other.

#### 3.2.4. Consistency Between the Semi-Markov Formulation and the Implemented Recursion

The aircraft trajectory is modelled as a spatially constrained inhomogeneous semi-Markov process, whereas Equations (9)–(11) iterate an ordinary transition matrix. Earlier versions of this paper left the relation between the two implicit, and the two are not in fact interchangeable unless stated with care. This subsection gives the exact relation, states which specialisation is implemented, and identifies what the specialisation cannot represent.

Let the embedded jump chain of the semi-Markov process be the chain {*J_m_*} that retains only the grid occupied immediately after each grid change, with jump kernel *P* whose diagonal vanishes, and let $N\left(n\right)=\max\left\{m:\sum_{r=1}^{m}\tau_{r}\leq n\right\}$ be the renewal count generated by the residence-time law *F_g_* of Equation (4). The grid occupied *n* minutes after the anchor is *X_n_* = *J_N_*_(*n*)_, and its distribution is the dwell-weighted convolution of the jump-chain powers,

(14) $$\mathrm{Pr}\left(X_{n}=j|X_{0}=i\right)=\sum_{m=0}^{n}{\left[P^{m}\right]}_{\mathrm{ij}}\mathrm{Pr}\left(N\left(n\right)=m\right),\;P^{0}=I,n\in \left\{1,\ldots ,5\right\}$$

Equation (14) is the exact semi-Markov rollout: the horizon-indexed marginal is a mixture of jump-chain powers, weighted by the probability that exactly *m* grid changes have occurred within *n* minutes. Equation (11) is the geometric-dwell case of the same expression, and the derivation is one line. Let the one-minute continuation probability of grid *g*, that is, the probability that the aircraft is still inside *g* at the next sample, be the diagonal entry *T_gg_* of the matrix in Equation (9), and let the jump kernel of the semi-Markov process be $\tilde{P}$, whose diagonal vanishes. With *D* = diag(*T_gg_*) and *D*′ = diag(1 − *T_gg_*), the estimated matrix then satisfies $P=D+D^{\prime }\tilde{P}$, because the only way to remain in a grid for one further minute is not to jump. Expanding the *n*-th power row by row gives, for the row of the anchor grid, $\sum_{m}C\left(n,m\right){\left(1-T_{\mathrm{gg}}\right)}^{m}{T_{\mathrm{gg}}}^{n-m}{\left[{\tilde{P}}^{m}\right]}_{\mathrm{gj}}$, which is Equation (14) with the renewal count binomially distributed. Under this dwell law, the two rollouts are therefore not approximations of one another: they are the same quantity written in two ways, and the parameter that ties them is measured from the data rather than assumed.

The dwell time therefore does enter the computation, and it enters at three points that can be checked against the listing in Algorithm 1. (i) Estimation. The diagonal of *P* is a measured quantity, not a modelling assumption: the entry for grid *g* is the ratio of the one-minute self-transitions counted in the knowledge base to all transitions recorded from *g*, and the mean dwell *λ_g_* = 1/(1 − *T_gg_*) used in Equation (4) is obtained from it. (ii) Rollout. The recursion applied in Algorithm 1 is the operator of Equation (11) acting on the delta vector of Equation (10), which by the identity above is the dwell-weighted convolution of Equation (14) evaluated in closed form rather than term by term. (iii) Retrieval. The context-conditioned matrix is produced by the same counting estimator with the same diagonal convention, so the identity holds row by row for the retrieval matrix as well, and the dwell law reaches the ablation results and the reported hit rates through the same single quantity. The relation between the two dwell laws used in the paper is stated here explicitly, because they are different objects: Equation (4) is the continuous-time description, adopted for its maximum-entropy property, while the law that a one-minute transition matrix can carry is its discrete-time counterpart, geometric with the same first moment. The paper uses Equation (4) to state the model and the geometric law to evaluate it, and the two agree in the only quantity that the evaluation consumes.

The first consequence is that the semi-Markov model is instantiated by Equations (9)–(11) exactly rather than approximately: the state space is the grid set, the kernel is the context-conditioned transition kernel of Equation (5), and the dwell component survives in the diagonal of the transition matrix, which the estimator fills from counted transitions rather than from a distributional assumption. The second is that the residence-time distribution is not merely decorative: it fixes the value of *T_gg_*, and that value is what distinguishes the model from a fixed-step Markov chain, in which the diagonal would be an assumption rather than a measured first moment. The caveat is that the identity is a statement about the estimator, not about the aircraft. It shows that the implemented recursion is the exact rollout under the dwell law that the data imply at one-minute resolution; it does not show that a dwell law of a different shape would give the same numbers. For an over-dispersed dwell law with the same mean, the renewal count is no longer binomial, Equations (14) and (11) separate, and the size of that separation is not measured here. This is the precise sense in which the present implementation is semi-Markov: fully semi-Markovian in its state space and in its kernel, first-moment in its dwell.

Two limitations follow from this specialisation. First, because the estimator reads only the first moment of the dwell, grids whose dwell distribution is strongly over-dispersed at a fixed mean, crossed in a single step on some flights and occupied for many minutes on others, cannot be represented by a single diagonal entry. Second, evaluating Equation (14) explicitly makes the rollout cost grow with the number of jump-index terms retained, which is why the closed form of Equation (11) is used. Exposing the full dwell-resolved rollout, with per-grid estimation of Fg beyond its mean, is left to future work.

For the airspace semi-Markov trajectory evolution model, the core solution objective is to accurately characterise the time-varying grid state transition law, stably solve the state transition matrix, and further support multi-step probabilistic iterative deduction. The mainstream solving paradigms of the existing Semi-Markov Process (SMP) and Markov temporal prediction system can be divided into three categories: global statistical frequency solving, implicit fitting solving based on deep models, and single-retrieval adaptive solving. However, the above schemes all have inherent mechanism defects in the scenario of minute-level tactical trajectory prediction for aircraft, and cannot simultaneously meet the multiple constraints of tactical prediction tasks, including lightweight inference, non-degenerate deduction, context adaptiveness, and interpretable airspace semantics. Based on this, we propose a targeted efficient hybrid retrieval Markov solving mechanism in the following section.
**Algorithm 1:** RAG-Hybrid Algorithm$\mathrm{Input}:\;\mathrm{Current}\;\mathrm{grid}\;g_{t},\;\mathrm{historical}\;\mathrm{grid}\;\mathrm{sequence}\;H_{1:t}\;=\;(g_{1},g_{2},\ldots ,g_{t}),\;\mathrm{Knowledge}\;\mathrm{Base}\;\mathrm{KB}(\mathrm{FAISS}\;\mathrm{index}\;+\;{\mathrm{text}}_{\mathrm{map}}\;+\;{\mathrm{pair}}_{\mathrm{freq}}\;\mathrm{edge}\;\mathrm{statistics}),\;\mathrm{global}\;\mathrm{transition}\;\mathrm{matrix}\;M_{\mathrm{global}},\;\mathrm{number}\;\mathrm{of}\;\mathrm{prediction}\;\mathrm{steps}\;n_{\mathrm{future}}$ $\mathrm{Output}:\;\mathrm{Multi}\text{-}\mathrm{step}\;\mathrm{prediction}\;\mathrm{distribution}\;P=\left\{D_{1},D_{2},\ldots ,D_{n_{\mathrm{future}}}\right\},$ where each *D_i_* = {*grid_id_*}:*probability* Parameters: *k* = 30, *w* = 16                    # FAISS retrieval size and retrieval window *D* = *n*_future_ + 1          # BFS expansion depth (hops) b = 5                               # Number of top-probability successors retained per grid per hop *α* = 0.5                  # Laplace pseudocount for transition-matrix extraction *w_r_* = 0.6                      # Fusion weight of the retrieval matrix against the global matrix *σ* = 0.6                  # Gaussian kernel bandwidth, in units of cell edge length Step 1: Direct Grid Lookup (BFS Hard Matching) 1: *out_edge_*←*KB* ∗ *pair_freq_* *#*   Precomputed: {*G_from_* → *Counter*{*G_to_*: *occurrence count*}) 2: *visited* ← {}, *frontier* ← {*g_t_*} 3: *C_direct_* ← [] 4: for *d* = 1 to *expand_depth_* do 5:        *next_frontier_* ← {} 6:        for *G* in *frontier*/*visited* do 7:             *visited* ← *visited* ∪ {*G*} 8:               *neighbors* ← *out*_*edges*.*get*(*G*) 9:               if *neighbors* is empty then continue 10:                *total* ← *Sum*(*neighbors*.*values*( )) 11:               *top* ← *TopK*(*neighbors*, *expand*_*topk*, *sorted by count*) 12:           *text* ← *Format*(*“Grid* {*G*} *transition probability*: →{*G*1}:{*n*1/*total*}%, →{*G*2}:{*n*2/*total*}%, …*”*) 13:               *C_direct_*.*append*(*text*) 14:               *next_frontier_* ← *next_frontier_*∪{*G′ for* (*G′*,_) *in top*} 15:         end for 16:       *frontier* ← *next_frontier_* 17:       if *frontier* is empty then break 18: end for # Scope of the deterministic channel: all out-edges of *g_t_* recorded in the knowledge base, within depth D and width b, are placed in the context irrespective of the embedding. This is a knowledge-base support guarantee, not a guarantee about the realised future cell. # Where the dwell time enters: the out-edge statistics precomputed in line 1 give the row diagonal *T_gg_*, the one-minute continuation probability of each grid, from which the mean residence time of Equation (4) is *λ_g_* = 1/(1 − *T_gg_*). With this measured diagonal, Markov-Iterate (line 25) evaluates the geometric-dwell case of the semi-Markov rollout of Equation (14) exactly rather than approximately. Step 2: FAISS Similarity Retrieval (Auxiliary Soft Supplement) 19: *q_vec_* ← Grid-Aware-Embed (*H*, focal = $“\mathrm{last}”,\;\sigma \;=\;0.6,\;w_{f}\;=\;3)$

20: $C_{{\mathrm{faiss}}_{\mathrm{raw}}}\;\leftarrow \;\mathrm{FAISS}\text{-}\mathrm{Search}(q_{\mathrm{vec}},\;k\;=\;30)$
21: $C_{\mathrm{faiss}}\;\leftarrow \;\mathrm{Dedup}(C_{{\mathrm{faiss}}_{\mathrm{raw}}}$, *against* = *C_direct_*) # Remove texts duplicated with direct lookup results Step 3: Hybrid Context Concatenation 22: *C_hybrid_* ← *Concat*([*Direct Exact Matching*] + *C_direct_*, *C_faiss_*)   #Direct matching texts placed in front to prioritise authentic statistics in downstream matrix extraction Step 4: Matrix extraction, blending and probabilistic inference 23: *M_rag_* ← Extract-Matrix-Enhanced(*C_hybrid_*, *α* = 0.5) 24: *M_ensemble_* ← Blend(*M_rag_*, *M_global_*, *w_r_* = 0.6) $25:\;[D_{1},D_{2},\ldots ,D_{n_{\mathrm{future}}}]$ ← Markov-Iterate(*g_t_*, *M_ensemble_*, *n_future_*) $26:\;\mathrm{return}\;[D_{1},D_{2},\ldots ,D_{n_{\mathrm{future}}}]$


## 4. RAG-Hybrid Algorithm Design and Analysis

### 4.1. Key Modules of the Proposed Algorithm

Traditional global Markov models adopt fixed, statistically global transition distributions that are independent of historical flight trajectories. Such methods fail to characterise the path-dependent transition characteristics of aircraft flight, namely that the same airspace grid may lead to entirely different subsequent flight directions given different incoming trajectory contexts. This context-free modelling framework severely limits the short-term accuracy of minute-level tactical trajectory prediction. To address the poor adaptability and missing local airspace semantic information of global statistical models, the retrieval-augmented paradigm is introduced for trajectory evolution modelling. Its core principle is to dynamically match similar historical flight patterns from a pre-established airspace prior knowledge base using the current short-term trajectory as a query. A context-constrained local transition matrix *T^rag^* is adaptively constructed to compensate for the inability of global models to adapt to real-time flight situations, thereby significantly improving the accuracy of short-step tactical prediction. This paper establishes two core foundational modules supporting the proposed RAG-Hybrid models, namely the dynamic FAISS knowledge base with Grid-ID-aware spatial vectorisation and progressive retrieval-augmented schemes.

Before describing the modules, it is worth stating precisely what the retrieval layer is expected to change, because the reported results show that the answer is narrower than the phrase retrieval-augmented generation suggests. A global Markov model estimates one transition row per grid, averaged over every flight that has ever passed through that grid. The retrieval layer replaces that row, for one query, by a row estimated from the subset of historical transitions whose surrounding context resembles the query. Two consequences follow. First, the ranking of successors can change relative to the global row, because the context-conditioned counts are drawn from a different and smaller sample; this is the operational meaning of context conditioning and is what Equation (5) expresses. Second, the support of the row, which successors are considered at all, is no longer limited to what stochastic retrieval happens to recall. Retrieval therefore acts on the candidate set rather than on the identity of the first-ranked candidate: because the first-ranked candidate is the argmax of a matrix that is blended with the global matrix through *W_r_*, the retrieval layer can shift probability mass between candidates without reordering the head of the distribution whenever the blended row remains dominated by the same mode. How much of this expectation the measurements bear out is a separate question from how the mechanism works: the mechanism fixes where the retrieval layer can act, and the measurements decide by how much it does.

(1) Dynamic FAISS Knowledge Base and Grid-ID-Aware Spatial Vectorisation

To achieve efficient retrieval and matching of airspace prior information, a FAISS retrieval knowledge base is offline constructed to integrate three types of heterogeneous airspace knowledge: grid-level airspace traffic heat distribution, normalised airway high-frequency transition triples, and grid-based Markov out-edge transition statistics. During online inference, a temporal fragment with window length *w* truncated from the tail of the historical trajectory is adopted as the query feature, and top-*k* retrieved similar priors are concatenated to form the final contextual set *C*. The IndexFlatIP structure is utilised for retrieval. After *L*_2_ normalisation, the inner product of vectors is equivalent to cosine similarity:

(15) $$\mathrm{sim}\left(q,x\right)=\frac{\phi \left(q\right)\cdot \phi \left(x\right)}{\left\|\phi (q)\|\|\phi (x)\right\|}$$

Retrieval performance is fundamentally determined by the representation capability of the vectorisation function *ϕ*(·). Conventional character hashing encodes text based purely on character matching while ignoring airspace spatial topological correlations. Spatially adjacent grids are treated as irrelevant due to different string identifiers, resulting in the failure to recall spatially neighboring valid priors. To solve this issue, this paper proposes a grid-ID-aware spatial vectorization strategy. Each grid token in the trajectory text is parsed into 2D spatial coordinates (*x*,*y*) = (*i_lon_*,*j_lat_*). An isotropic Gaussian kernel is superimposed on the *M* × *N* grid plane and flattened into a spatial vector:

(16) $$\phi (\mathrm{text}{)}_{\left[x^{\prime },y^{\prime }\right]}=\sum_{\left(x,y)\in G(\mathrm{text}\right)}\omega (x,y)\exp\left(-\frac{\sum {\left(x^{\prime }-x{)}^{2}+(y^{\prime }-y\right)}^{2}}{2\sigma^{2}}\right)$$

where *G*(*text*) denotes the set of grid coordinates contained in the input trajectory text, and *σ* represents the Gaussian kernel bandwidth. In this paper, *σ* is set in units of cell edge length, and a sharp kernel with *σ* is adopted to ensure that the focal grid dominates the feature representation while allowing slight feature diffusion to adjacent grids, balancing unique grid identity and spatial neighborhood modeling and avoiding erroneous prior injection caused by over-smoothing. Meanwhile, a focal weighting factor *ω* is introduced to assign higher weights to the current critical grid at the end of the query sequence, realizing strong vector-space alignment between query trajectories and knowledge base priors. Compared with character hashing, the proposed spatial vectorization delivers physically meaningful spatial airspace similarity and possesses superior generalization capability for spatially adjacent unseen grids.

(2) Progressive Retrieval-Augmented Schemes

Based on the aforementioned retrieval framework, a basic retrieval-augmented model named RAG-Original is first developed to compensate for the context deficiency of global Markov models. With a retrieval window *w* and top-*k* retrieved samples, RAG-Original constructs the local transition matrix *T^rag^* solely from Markov transition records retrieved from contextual priors, enabling dynamic adaptive modeling of trajectory transition patterns. Nevertheless, this basic scheme suffers from inherent structural defects. FAISS vector retrieval involves stochastic missing detection. If the transition priors corresponding to the current grid *g_t_* are not recalled, the corresponding row in *T^rag^* will be missing. The inference process degenerates to fixed self-loop transitions, and the model degrades to persistence prediction. Consequently, the Top-3 and Top-5 probabilistic distributions collapse into single-point outputs, disabling the probabilistic prediction capability.

### 4.2. Description of the RAG-Hybrid Algorithm

To address the omission failure of stochastic matching and the uncertain local subgraph that existing retrieval-augmented methods acquire, this paper proposes a dual-channel hybrid retrieval algorithm (RAG-Hybrid). The design target is narrow and stated in advance: make the recall of the anchor grid’s recorded out-edges independent of the similarity metric, so that the local transition row is no longer contingent on whether vector search happens to return the matching records. The guarantee this yields is over the knowledge base, not over the aircraft’s future motion, and it is bounded by the expansion depth and per-hop successor width of the traversal; Both the scope and the residual are measured in the experiments below. The detailed algorithm design and implementation pipeline are presented below.

Algorithm 1 illustrates the overall prediction pipeline for minute-level grid-based trajectory forecasting implemented by the RAG-Hybrid algorithm. Its core idea is to combine deterministic breadth-first search (BFS) local subgraph expansion with FAISS vector-similarity retrieval, integrating hard spatial priors read directly from recorded transition counts with soft context drawn from analogous historical trajectories. The task is decomposed into knowledge-base lookup, context construction, transition-matrix fusion and multi-step power-iteration inference. The final context for transition-matrix construction is produced by deduplicating and concatenating the outputs of the two channels, with the deterministic channel placed first. What the deterministic channel guarantees is precise, and conditional. For the anchor grid *g_t_*, every successor that is present in the knowledge base and reachable within expansion depth *D* through the per-hop successor width *b* is placed in the context irrespective of the similarity metric, so recall over the recorded out-edges does not depend on the embedding. It does not guarantee that the aircraft’s realised next grid is present in the knowledge base. If a transition has never been observed in the training flights, no retrieval strategy can return it, and the prediction falls back on the global prior. The measured coverage, namely 93.47% of test grids and 79.38% of test transition edges, is precisely the scope of the guarantee; the remaining 20.62% of realised test transitions are unseen transitions that lie outside it. The traversal is in addition truncated at depth *D* and width *b*, so successors reachable only through low-frequency edges are excluded by construction. The Top-K hit rate and the probabilistic prediction error are adopted as performance metrics to evaluate the context quality and the overall forecasting performance.

As shown in Algorithm 1, the RAG-Hybrid algorithm mainly consists of three processes: dual-channel prior retrieval, context-aware transition-matrix construction, and semi-Markov power-iteration prediction. Lines 1–18 (Direct Grid Lookup Phase): Starting from the current grid *g_t_*, breadth-first search is executed over the knowledge-base out-edge statistics. Within predefined expansion depth *D*, top-b high-probability successor grids are collected at each hop to build deterministic local transition texts. Lines 19–21 (Similar-Trajectory Retrieval Phase): The trailing window of input historical trajectory is encoded by the grid-ID-aware spatial vectorizer to construct the query vector, and Top-k similar prior records are returned via FAISS inner-product search. Lines 22–26 (Context Fusion and Probabilistic Inference Phase): Merge and deduplicate the outputs of two retrieval channels, putting direct-lookup results in priority position. Laplace smoothing is applied to extract the local retrieval-based transition matrix ${\mathrm{rag}}_{T}$, which is further blended with the global statistical transition matrix to obtain the final transition matrix *T*. Since pure vector retrieval cannot guarantee the coverage of current-grid transition records, the deterministic BFS lookup module is introduced so that recall over the recorded out-edges does not depend on the similarity metric and prevents prediction collapse to persistence. The initial probability vector is initialized according to the current grid state, and multi-step Markov power iteration is performed to generate n-step probabilistic prediction results (Lines 23–25).

Three functions called in Algorithm 1 are defined here, since earlier versions of this paper referred to them only by name. Extract-Matrix-Enhanced(*C*_hybrid_,*α*) parses the retrieved transition statements into a weighted directed multigraph, converts the counts of each source grid into a row-normalised transition probability vector, adds *α* pseudo-counts to every entry of every row that the context populates, and re-normalises the row; *α* is therefore a Laplace pseudocount in the sense of [13] and is not a Gaussian quantity. Blend(*M*_rag_,*M*_global_,*w_r_*) forms the convex combination *M* = *w_r_* · *M*_rag_ + (1 − *w_r_*) · *M*_global_ and re-normalises each row, taking rows that the retrieval context leaves empty directly from *M*_global_. Markov-Iterate(*M*,*g_t_*,*n*) forms the delta initial vector of Equation (10) at *g_t_* and applies the iteration of Equation (11) *n* times. In Algorithm 1, these correspond to lines 23, 24 and 25 respectively, and the earlier cross-references to ‘Steps 2–4’ and ‘Lines 4–19’ have been removed because they referred to this algorithm itself.

## 5. Discussion

### 5.1. Experimental Settings

As for dataset and gridded airspace, ADSB flight trajectory data are adopted for validation [46]. Raw trajectories are resampled at a 1-min interval and further discretised to generate flight sequences over gridded airspace. The experimental airspace covers Central-South China, spanning 104–118° E in longitude and 18–36° N in latitude. The grid resolution is set to 0.25° × 0.25°, yielding a total of 4032 grid cells (56 × 72). The evaluation set contains 62 flights with sequence lengths ranging from 11 to 149 steps and an average length of 82.2 steps. Within the test set, there are 919 unique grid cells and 1993 state transition edges. The self-loop ratio reaches 59.65%, where 3004 out of 5036 transition steps stay inside the same grid. Since the sampling interval is fixed at one minute, the *n*th step in the sequence corresponds to the flight position *n* minutes ahead in the future.

As for the FAISS knowledge base, we construct an offline retrieval repository that integrates three heterogeneous airspace priors, including traffic heat distribution, regular airway transition records, and grid-based Markov transition statistics. Two embedding methods are supported, namely conventional character hashing and Grid-ID-aware spatial vectorisation. The knowledge base is trained on an extended dataset of 290 flights, while model evaluation is conducted on an independent test set containing 62 real flight trajectories. The established library covers 2484 unique grids and 9913 transition edges with a self-loop ratio of 67.88%. The test set includes 919 valid grids, 1993 transition edges, and 5036 transition samples. Quantitative statistics verify high coverage between the knowledge base and test data. Specifically, 93.47% of test grids, all one-minute prediction anchor grids, and 79.38% of test transition edges can be retrieved from the established base. Such high coverage demonstrates that retrieval missing is not the dominant factor causing prediction degradation, which supports the subsequent experimental analysis.

As for the evaluation protocol, each flight trajectory is divided into historical observation segments and future prediction segments. For multi-step prediction tasks, earlier trajectory segments are adopted as model inputs, and the final grid state is taken as the prediction target. Three evaluation paradigms are used, and their distinction is stated here explicitly because earlier versions of this paper left it implicit and because it determines how the reported confidence intervals must be read. (i) Horizon-specific anchors. For each flight and each horizon *k* in {1, …, 5}, an anchor *g*_t_ is drawn such that the target *g*_t+k_ lies inside the same flight, giving 62 anchors per horizon. The single-step statistics and the per-horizon columns are computed on these anchors, one horizon at a time. (ii) Fixed-anchor rollout. A single anchor per flight is used, and the distribution is propagated for five steps, so the five horizon values share an initial state and are serially correlated by construction; this protocol isolates the behaviour of the rollout itself, and it underpins the corresponding experimental results. It is not used for any pooled statistic. (iii) Aggregated multi-horizon metrics, obtained by concatenating the per-horizon results of (i), giving 310 valid test samples in total. These 310 samples are not independent, since at most five of them come from one flight, and the resolution of the pooled comparisons is derived accordingly below. For rigorous comparison, all performance analyses are conducted under identical prediction horizons rather than across different time scales.

As for statistical sufficiency, the size of the test set is a property of the data rather than a design choice, and its consequences are quantified here rather than asserted. The 62 test flights are the complete, gap-free trajectories in the public broadcast ADS-B archive [46] that satisfy the continuity and duration requirements of the fixed one-minute sampling grid (Section 3.1.1); enlarging the set would require a longer archive period, not a different protocol. Because the unit of analysis is the prediction sample rather than the flight, counts are reported at both levels, and the resolution of the comparison is derived from them. Under protocol (i), the single-step statistics rest on 62 anchors, one per flight, which are independent across flights; for a proportion near 0.71, the binomial standard error is 0.058, and the 95% half-width is 0.113. Under protocol (iii), the pooled statistics rest on 310 samples, but at most five of them come from one flight, so independence cannot be assumed; with 5.0 samples per flight and an intra-cluster correlation of *ρ*, the design effect is 1 + (5 − 1)*ρ*, and at *ρ* = 0.3 it equals 2.2, giving an effective sample size of 141 and, for a proportion near 0.745, a 95% half-width of 0.072. The corresponding bound at the +5 min horizon, where the hit rate is near 0.565, is 0.124. A single reporting rule is therefore applied throughout the paper: a difference between two hit rates is described as resolved only if it exceeds the bound for its protocol, and is otherwise reported as a trend or as unresolved at this sample size. Two claims made in earlier versions of this paper fail that rule and have been corrected or withdrawn: the pooled Top-5 difference against the fair global-Markov baseline (−0.0096) and the Top-3/Top-5 gain at the +1 min horizon (+0.0484) are both below threshold, whereas the effect of removing the deterministic lookup channel (+0.0936 pooled, +0.1935 at the +5 min horizon) exceeds it and is the component effect that this study establishes.

As for the experimental fairness strategy, previous RAG-based methods suffer from severe data leakage. Early versions improperly adopted transition statistics calculated from the evaluation set for global Markov fusion, which introduced implicit prior information and caused overestimated performance. In this paper, all RAG variants adopt transition matrices trained purely on extended training data to completely avoid data leakage. Global Markov with fair training settings serves as a unified baseline for comparative experiments. Another Global Markov variant utilising test-set statistics is regarded as the empirical performance upper bound, which is only used for ceiling reference and excluded from formal comparison.

As for data partitioning and tuning protocol, three statements are needed to establish that the tuning of RAG-Hybrid was not driven by the test results. First, the partition is disjoint at flight level: the 290 knowledge-base flights, the subset used for hyperparameter selection and the 62 test flights are mutually exclusive sets of trajectories, so no flight contributes both a retrieval neighbour and a test sample. Second, hyperparameter selection used the validation subset only: the retrieval size *k*, the smoothing coefficient *α*, the fusion weight *w_r_*, the BFS expansion depth *D* and the successor width *b* were chosen on that subset, and none of the test comparisons was used to select them. Third, the deep-learning baselines were trained and tuned under a protocol matched to this one—the same flight-level partition, the same exclusion of selection flights from the retrieval knowledge base, early stopping on validation Top-1 with learning-rate decay, and the same evaluation on the 62 test flights with the same anchors and the same Top-K protocol. No separate architecture search was performed: the models are standard published forms [47,48,49] with a grid-classification output layer, and they are included to characterise the behaviour of parametric learners in this sample regime rather than to establish the best achievable deep baseline. One asymmetry remains and is stated rather than concealed: the deep models use a single fixed random seed (42), so their reported values are point estimates rather than seed-averaged means, whereas the retrieval framework is deterministic and requires no seed averaging. Repeating the training with multiple seeds, and comparing the resulting spread against the bounds derived above, is left to future work.

As for statistical verification, the tests are chosen to match the structure of the data. The paired Wilcoxon signed-rank test is applied to continuous metrics such as prediction probability and distance error, and the McNemar exact test to pairwise comparison of Top-K hit rates on the same anchors, so that each comparison is made within anchors rather than between marginal proportions. Bootstrap resampling with 2000 iterations generates 95% confidence intervals for hit-rate curves and accuracy-decay trends; the resampling unit is the flight rather than the sample, so the five samples contributed by one flight enter or leave a replicate together. This is the clustering-corrected form of the interval and is wider than the naive per-sample bootstrap by a factor of about 1.5 at the intra-cluster correlation used above. The Fisher exact test is retained for cross-model comparison on subsets too small for an asymptotic test. Where a reported difference falls below the resolution bound derived above, the interval is still reported, but the difference is not interpreted as evidence of superiority.

### 5.2. Comparative Methods

Based on grid-coded trajectory representation, the proposed RAG-Hybrid method combines three components: a deterministic BFS lookup over recorded grid out-edges, FAISS-based soft vector retrieval, and global-Markov fusion. The symbols are used consistently throughout the paper as follows: *k* is the FAISS retrieval size, i.e., the number of prior records returned by one query; *w* is the retrieval window in minutes; *α* is the Laplace pseudocount added during transition-matrix extraction, and is the same quantity as the *β* of Equation (12); *w_r_* is the fusion weight of the retrieval matrix against the global matrix; *σ* is the Gaussian kernel bandwidth of the grid-ID-aware vectorisation, expressed in units of cell edge length; *D* is the BFS expansion depth in hops; *b* is the number of highest-probability successors retained per grid per hop; and *w_f_* is the focal weight applied to the anchor grid. In earlier versions of this paper, the expansion depth and the successor width were respectively mislabelled as a ‘Gaussian kernel dimension *D*’ and a ‘kernel bandwidth *b*’, and the bandwidth value was omitted; these names and values are corrected here and used consistently from this point on.

Several statistical baselines are selected for comparison. Persistence serves as the trivial lower-bound predictor that always retains the current grid for subsequent prediction, representing the self-loop baseline [7]. Constant velocity dead-reckons the aircraft by the displacement observed over the preceding one-minute sample and predicts the cell containing the dead-reckoned position. It is the natural non-parametric motion-model baseline for a grid predictor and the most accurate point predictor in this study, attaining the highest +1 min Top-1 rate of all ten methods (0.8065) together with the smallest mean absolute error (4.90 km). Two properties of its construction must be kept in view. First, it is deterministic: it returns a single cell, so its Top-3 and Top-5 rates equal its Top-1 rate, exactly as for Persistence. Second, it is the only baseline that consumes the raw broadcast coordinates rather than the discretised grid sequence, because a velocity vector cannot be estimated at the 0.25° quantisation; every other method, including the proposed framework, sees only a sequence of grid identifiers. Like Persistence, it uses no transition statistics and therefore cannot benefit from the knowledge base. Global Markov (fair) is a first-order Markov model whose transition matrix is learned from the extended training dataset without data leakage, following the conditional Markov trajectory modelling scheme of [10]. Global Markov† adopts transition statistics computed directly from the evaluation set and therefore delivers an empirical upper-bound performance; it is excluded from formal comparison and is shown in the figures only as a ceiling reference. RAG-Original is the preliminary prototype of the retrieval-augmented approach, adopting a small retrieval scale (*k* = 8, *w* = 8) together with character-hash embedding.

The deep-learning baselines are the multilayer perceptron (MLP) [47], long short-term memory (LSTM) [48] and gated recurrent unit (GRU) [49] networks. All three are trained on the identical extended training set of 290 flights and evaluated on the independent test set of 62 real ADS-B trajectories, under the partitioning and tuning protocol set out above. Model training uses early stopping and learning-rate decay, with the random seed fixed at 42.

An ablation study is further designed for RAG-Hybrid. Five core components are removed one-by-one in separate variant experiments, namely the direct grid lookup branch, FAISS soft retrieval branch, Laplace smoothing module, global Markov fusion module, and GridID-aware spatial vectorisation module.

### 5.3. Experimental Results

#### 5.3.1. Ablation Study

(1) Component-Level Ablation Study

**Figure 2:**
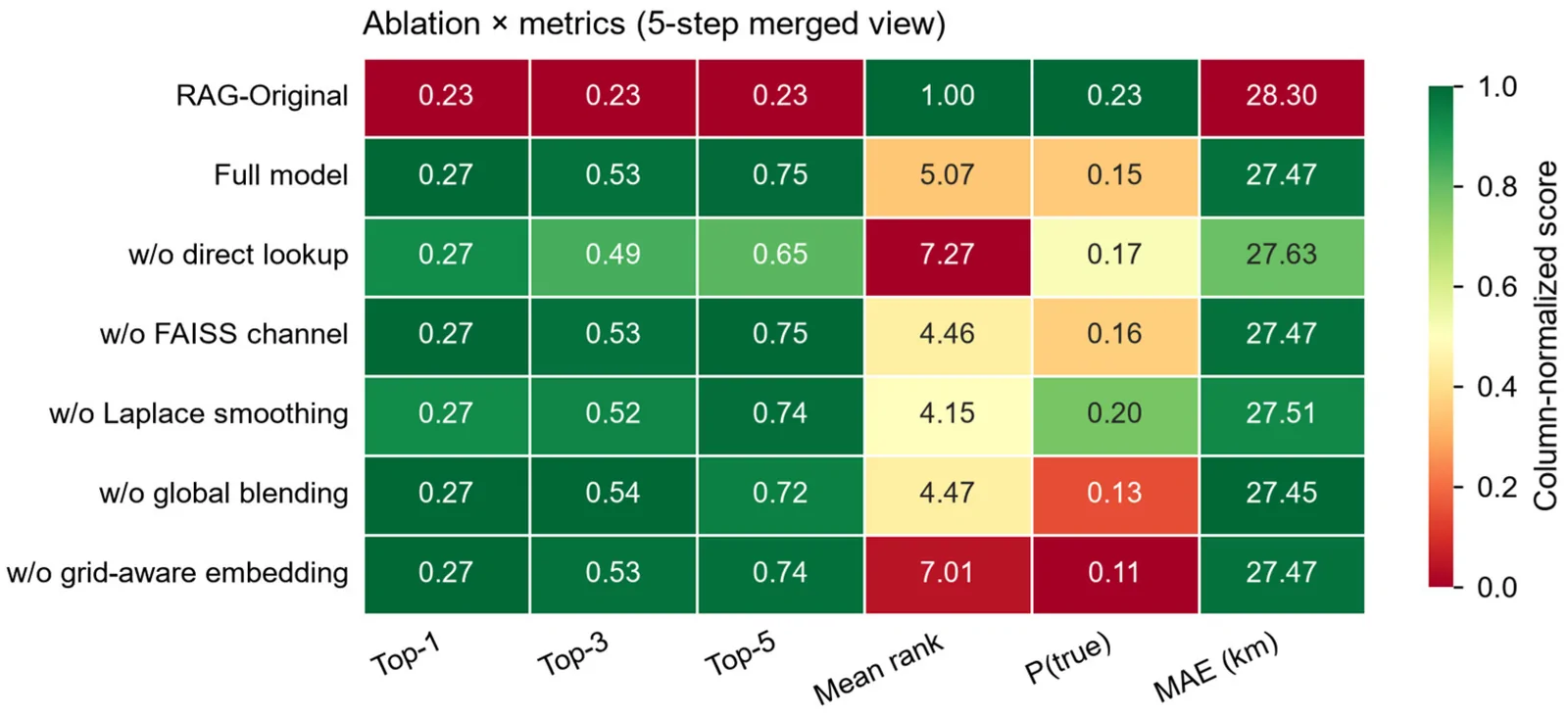
Heatmap of ablation conditions versus evaluation metrics.

**Figure 3:**
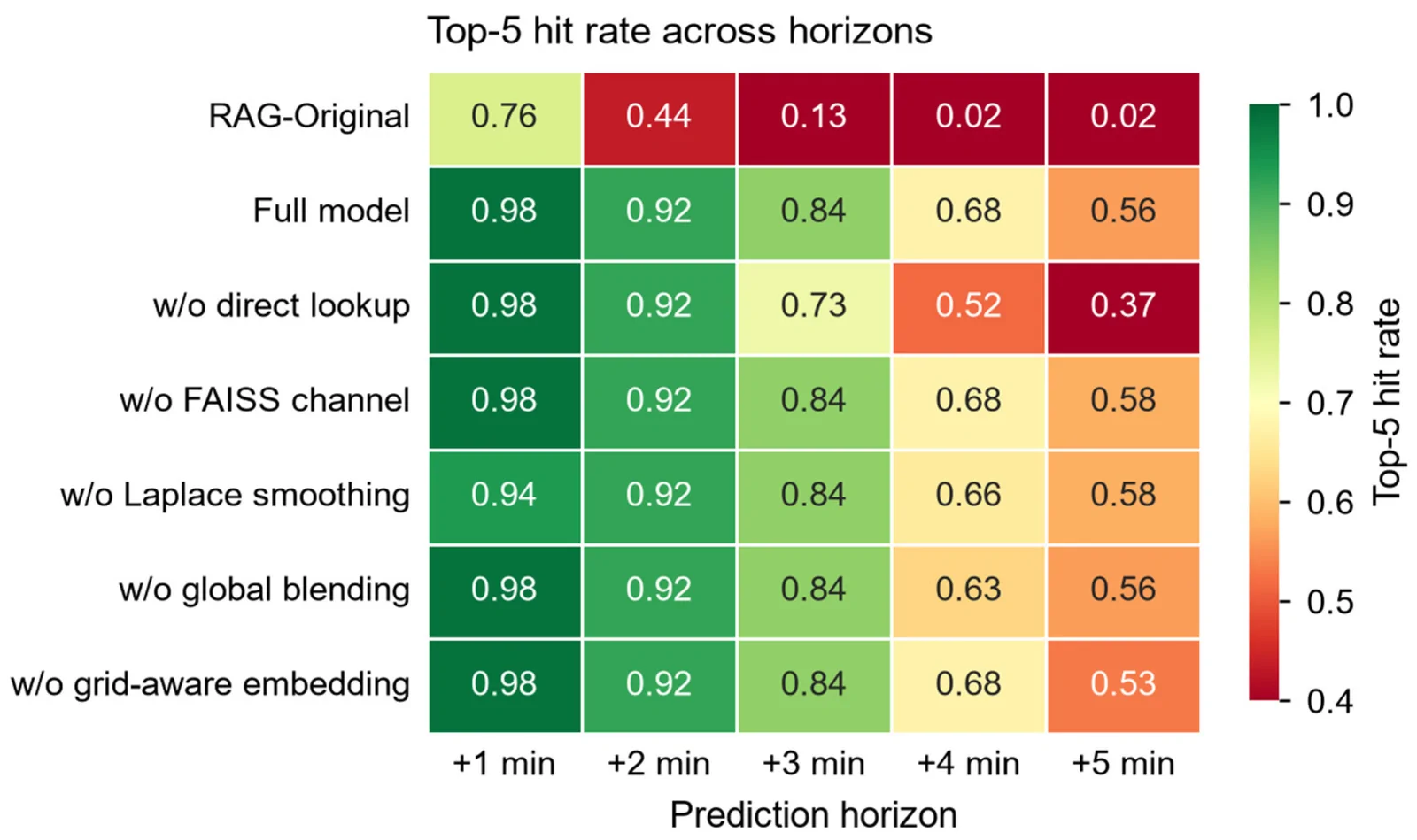
Top-5 hit rate at different prediction horizons for each ablation variant.

**Figure 4:**
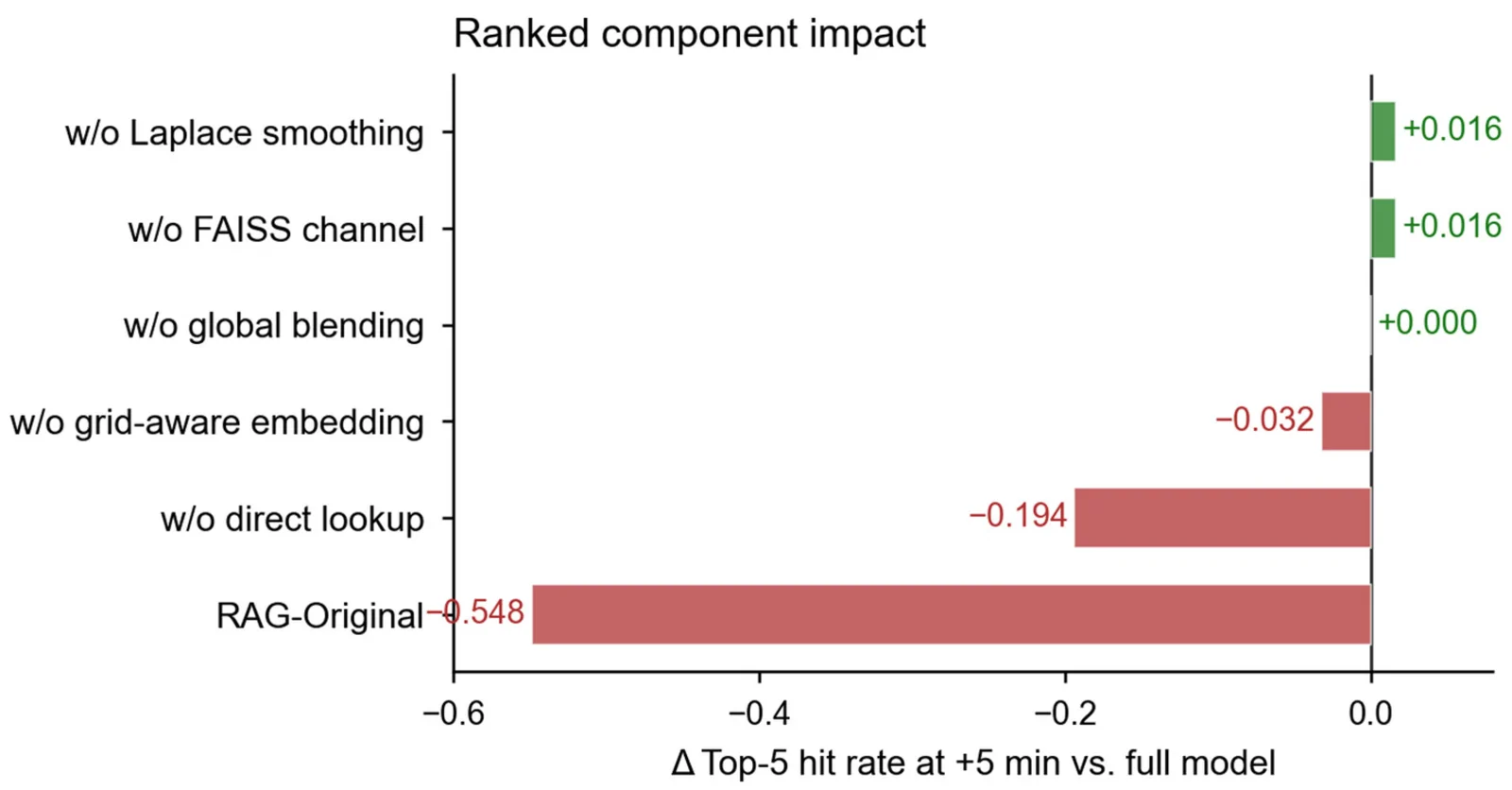
Ranked component impact: changes in the +5-min Top-5 hit rate for each ablation variant relative to the full model.

To quantitatively investigate the individual contribution and functional mechanism of each module in the proposed RAG-Hybrid framework, comprehensive component-level ablation experiments are conducted. The pooled five-step quantitative results and full-horizon Top-5 performance of all ablation variants are presented in Table 2 and Table 3, respectively, while the visualised metric heatmap, horizon-wise performance curves, and component impact ranking are illustrated in Figure 2, Figure 3 and Figure 4.

Several consistent conclusions can be drawn from the ablation results, and they are stated against the resolution bounds derived for this design rather than as qualitative judgements. First, the single-step decision is controlled by the calibrated Markov statistics rather than by retrieval. Removing the direct-lookup branch, the FAISS branch or the grid-ID vectorisation leaves pooled Top-1 at 0.2742, unchanged from the full model, whereas removing Laplace smoothing lowers it to 0.2710, a difference of 0.0032 that is far below the 0.072 pooled bound. The same module has the largest effect of any component at the +1 min horizon, where its removal lowers Top-1 from 0.7097 to 0.6774, a difference of 0.0323 that is likewise below the 0.113 bound for that protocol; the same quantity moves monotonically over a full sweep of the smoothing coefficient. The defensible reading of these numbers is therefore not that Laplace smoothing dominates single-step accuracy but that the single-step decision is stable under removal of every retrieval component, while smoothing is the only component whose removal moves the first-ranked output at all. The retrieval branches act on the multi-candidate set rather than on the first-ranked output.

Second, the deterministic direct-lookup branch is the core component that sustains the long-horizon forecasting superiority of RAG-Hybrid. As reported in Table 3, removing this module causes negligible performance variation within the first two minutes, but triggers severe performance degradation starting from the +3-min horizon. Specifically, the Top-5 hit rate drops sharply from 0.6774 to 0.5161 at +4 min and from 0.5645 to 0.3710 at the +5-min long-range horizon. Such a significant performance collapse does not appear in other ablation variants. With the extension of prediction steps, the aircraft’s reachable grid state space expands rapidly, and stochastic FAISS retrieval alone cannot stably recall low-frequency but valid spatial transition paths. The deterministic direct-lookup branch compensates for this instability and maintains the completeness of candidate sets over extended inference steps, which is what sustains its advantage over the parametric learners at the far end of the horizon.

Third, the FAISS soft-retrieval branch and the grid-ID-aware vectorisation act on posterior calibration rather than on candidate-set coverage, and their marginal contribution is not resolvable at the present sample size. As shown in Table 2, removing either module leaves Top-1 and Top-3 unchanged to four decimal places and changes pooled Top-5 by +0.0032 when FAISS is removed and by −0.0065 when the vectorisation is removed. At the +5 min horizon, removing FAISS raises Top-5 from 0.5645 to 0.5806, a difference of 0.0161, or five of the 310 pooled samples (Table 3). Every one of these differences lies below the 0.072 pooled bound derived for this design, so no directional conclusion is drawn from them. What the ablation does resolve is the effect on the confidence metric: removing FAISS raises *P*(true) from 0.1545 to 0.1562, removing the vectorisation lowers it to 0.1133, and removing global fusion lowers it to 0.1306. These are consistent with a role in shaping the calibrated distribution rather than the candidate set. The claim made in earlier versions of this paper, that the FAISS component contributes substantially to the overall performance of RAG-Hybrid, is therefore withdrawn. The correct statement is that FAISS and the grid-ID vectorisation place a physically meaningful similarity metric into the retrieval space and keep *P*(true) informative, that their removal is not detectable as a hit-rate effect at the resolution available here, and that a configuration without the FAISS channel is a legitimate lighter alternative which this paper now reports as such. The metric heatmap in Figure 2 and the horizon-varying Top-5 curves in Figure 3 show the same pattern, and Figure 4 ranks the component impact on the +5 min Top-5 hit rate.

(2) Vectorisation Ablation Study

**Figure 5:**
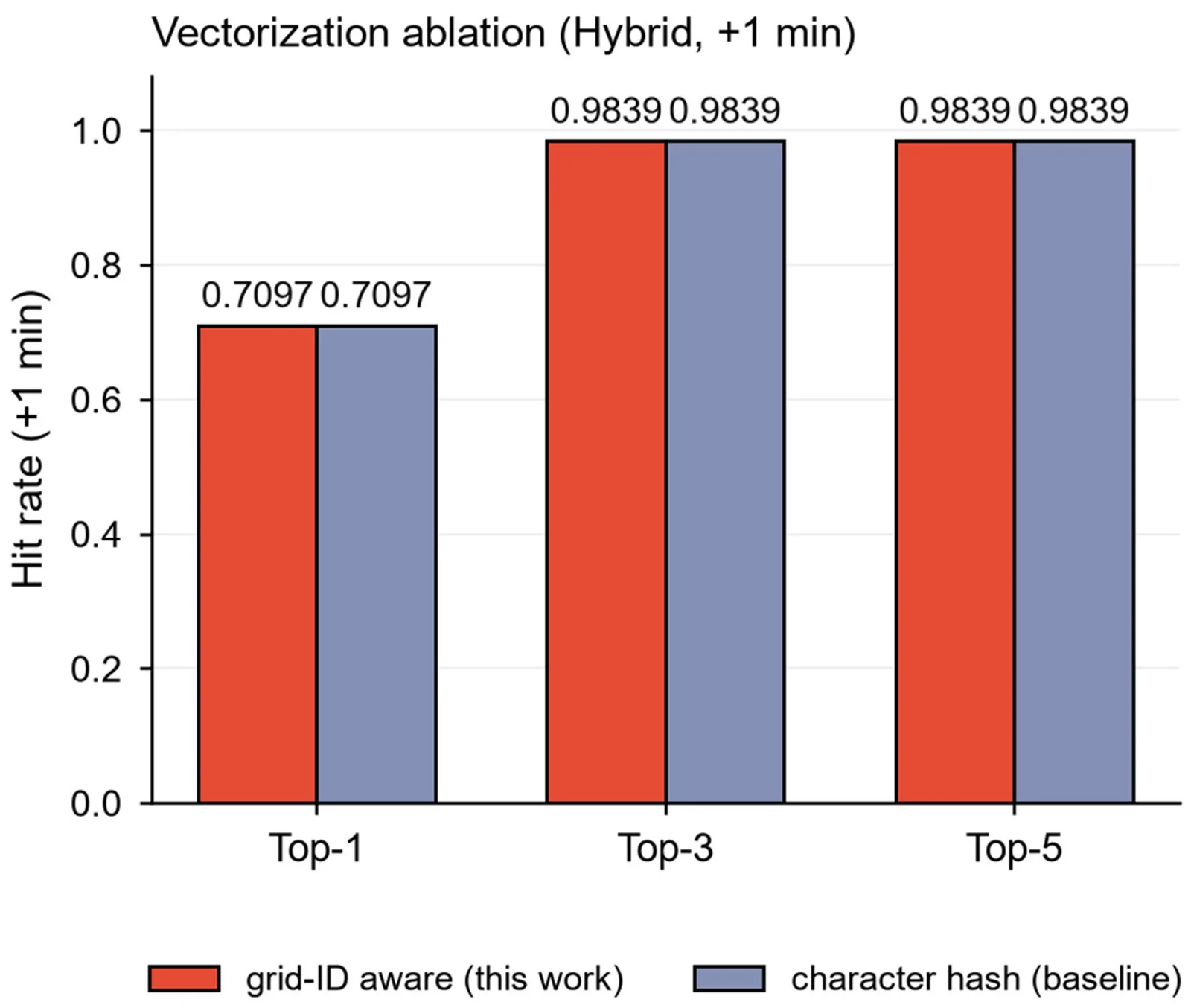
Vectorisation ablation: Grid-ID-aware vectorisation versus character hashing (1-min future prediction).

For the +1-min short-term prediction scenario, a further comparison is conducted between two embedding schemes: Grid-ID-aware spatial vectorisation and conventional character hashing. The ablation results are presented in Figure 5. Under the fair experimental setting with data leakage eliminated, both embedding schemes yield identical Top-1 (0.7097), Top-3 (0.9839), and Top-5 (0.9839) metrics. The underlying mechanism behind this phenomenon is that, under short-horizon conditions, the top-one prediction does not depend on embedding-based retrieval, and the prediction result remains dominated by the base Markov transition statistics. Vector embeddings only serve FAISS similarity retrieval. For single-step prediction tasks, the candidate set returned by retrieval does not interfere with the top-one output decision, and therefore spatial vectorisation cannot improve short-horizon prediction hit rates. If this module does introduce performance gains, its effect should manifest in the optimisation of the internal ranking of retrieved candidates under longer prediction horizons, rather than in directly improving single-step prediction accuracy.

#### 5.3.2. Parameter Sensitivity Analysis

The default hyperparameter settings are: FAISS retrieval size *k* = 30, retrieval window *w* = 16 min, Laplace smoothing coefficient *α* = 0.5, global-fusion weight *w_r_* = 0.6, Gaussian kernel bandwidth *σ* = 0.6 cell units, focal weight *w_f_* = 3, BFS expansion depth *D* = *n*_future_ + 1, which equals 3 for the five-step horizon used here, and per-hop successor width *b* = 5.

**Figure 6:**
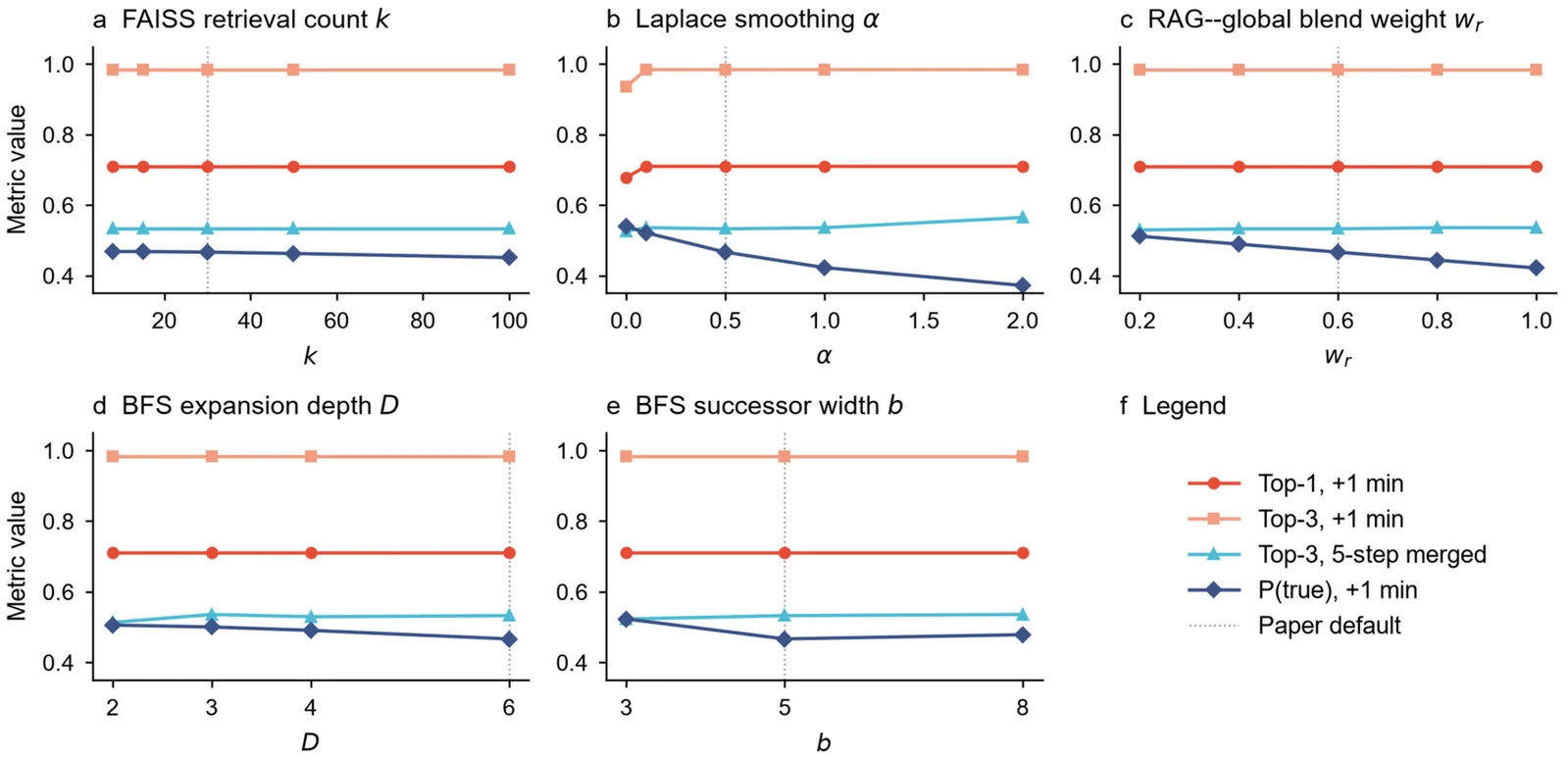
Hyperparameter sensitivity analysis of RAG-Hybrid.

To explore the robustness of RAG-Hybrid, a comprehensive hyperparameter sensitivity analysis was conducted by individually sweeping five core hyperparameters while keeping the remaining settings at their defaults: the FAISS retrieval size *k*, the Laplace smoothing coefficient *α*, the global-fusion weight *w_r_*, the BFS expansion depth *D* and the per-hop successor width *b*. The results are shown in Figure 6. One-step Top-1 prediction accuracy remains invariant across all tested values of *k*, *D* and *b*, maintaining a steady value of 0.7097; these retrieval-oriented hyperparameters induce only minor variations in the probabilistic confidence metric *P*(true). Among the five, only *α* affects deterministic Top-1 prediction appreciably, with the accuracy increasing from 0.6774 to 0.7097 as *α* rises from 0 to 2.0. That increase of 0.0323 lies below the 0.113 resolution bound of this design and is therefore reported as a monotone trend rather than as an established effect. Both *α* and *w_r_* impose clear and monotonically decreasing influences on *P*(true), jointly shaping the calibrated probabilistic distribution of the model output. These findings support a single reading: the retrieval branches do not participate in the final top-ranked decision, whereas smoothing and global fusion dominate the calibration of prediction confidence and the shape of the distribution. The Gaussian kernel bandwidth *σ* is not swept and is fixed at 0.6; sensitivity to it is left to future work.

#### 5.3.3. Spatial Performance Distribution

**Figure 7:**
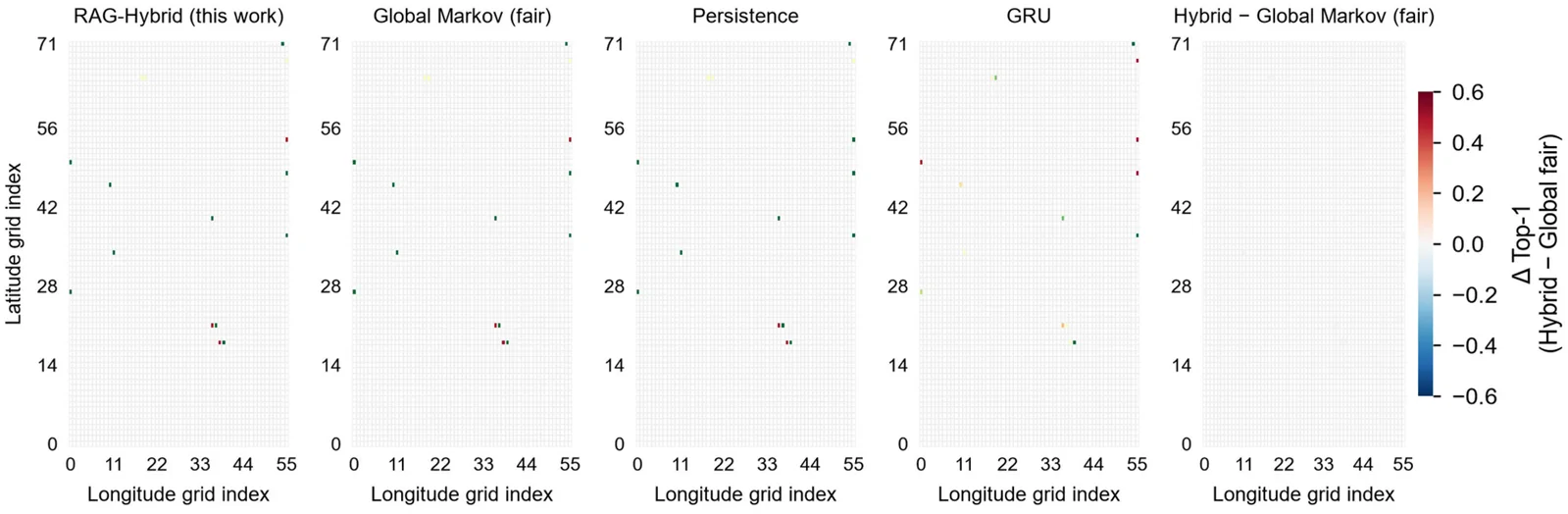
Spatial Top-1 hit rate distribution on a 0.25° × 0.25° grid.

To intuitively analyse the spatial generalisation capability, we aggregate flight test samples onto the standardised 0.25° × 0.25° gridded airspace and visualise the local Top-1 prediction accuracy across the entire 56 × 72 grid. The results indicate that RAG-Hybrid and the fair Global Markov baseline yield nearly identical optimal grid predictions for most test queries, leading to almost zero spatial difference throughout the airspace. The few residual non-zero grid discrepancies are exclusively distributed in sparse boundary regions, where valid flight samples are extremely limited. After masking grids with fewer than two valid samples, the remaining spatial deviations are confirmed to be random sampling noise rather than systematic structural differences caused by the retrieval mechanism. Comprehensive quantitative and visual results, including flight-level confidence calibration, time-varying heatmaps, multi-step accuracy decay curves with 95% bootstrap confidence intervals, and component ablation statistics, are collectively demonstrated in Figure 7 to support the overall performance analysis.

#### 5.3.4. Multi-Candidate Hit Performance at Successive Horizons

**Figure 8:**
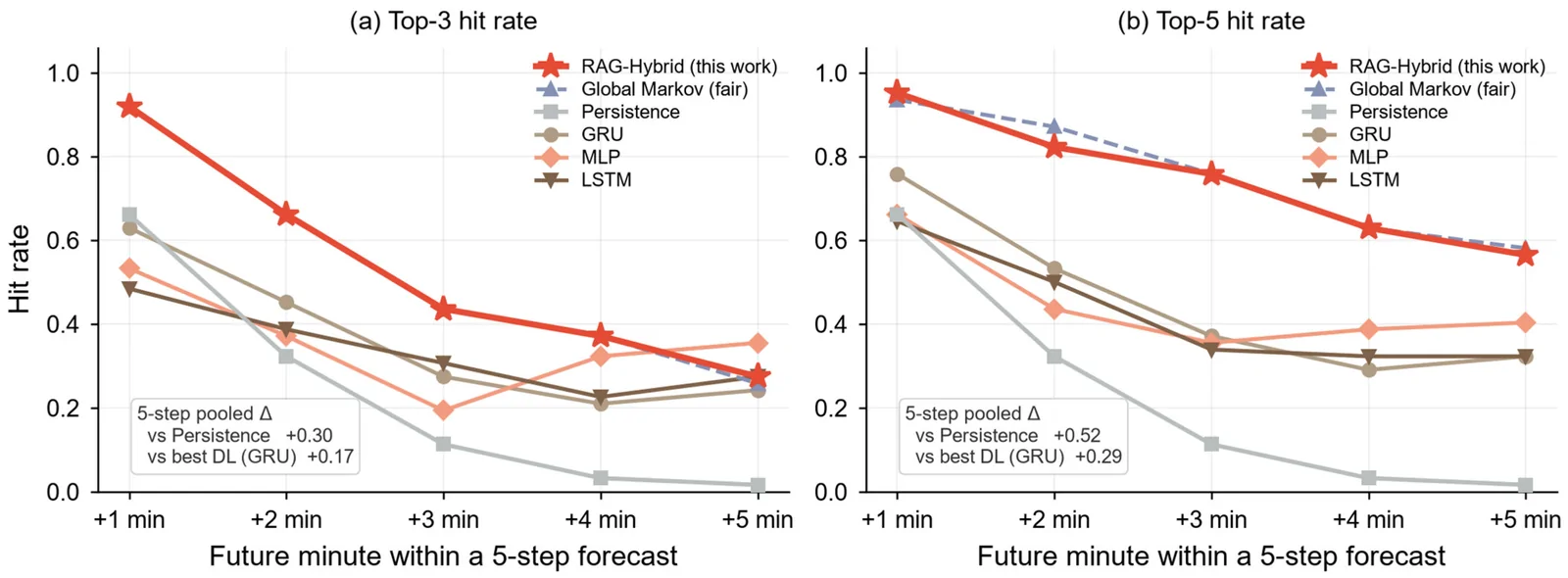
Variations of Top-3 (**a**) and Top-5 (**b**) hit rates with future minutes under five-step prediction starting from identical initial states.

Compared with the Persistence predictor, RAG-Hybrid achieves substantial performance gains, with improvements of 0.3033 for Top-3 hit rate and 0.5162 for Top-5 hit rate. Since Persistence always keeps the aircraft inside the current grid, its prediction performance degrades drastically for longer forecast horizons. The proposed method also yields systematic advantages over the three deep-learning baselines. Across the pooled five-step results, the Top-3 improvement ranges from 0.17 to 0.20, and the Top-5 improvement ranges from 0.29 to 0.32. This performance gap persists for every single future time step within the 5-min horizon, as illustrated in Figure 8. Figure 8 plots Top-3 and Top-5 hit rates against prediction lead time. The red solid curve with star markers for RAG-Hybrid stays above the curves of Persistence, GRU, MLP and LSTM at each forecast minute, while the grey-blue dashed curve of Global Markov (fair) nearly overlaps with our curve. The inset sub-box in the lower-left corner visualises the aggregated performance gain after pooling results over five prediction steps. Nevertheless, RAG-Hybrid shows very close performance to the fair Global Markov baseline. It delivers a marginal increment of 0.0033 on the Top-3 metric and a tiny decrement of 0.0096 on the Top-5 metric. Both deviations lie below the 0.072 pooled resolution bound of this design and are therefore reported as unresolved rather than as agreement: at this sample size, the study cannot distinguish the two methods on the pooled top-three and top-five metrics. Looking into per-step numerical results for each forecast minute from +1 min to +5 min, it can be observed that MLP attains a slightly higher Top-3 hit rate (0.3548) than RAG-Hybrid (0.2742) only at the final +5-min step. This isolated phenomenon is attributed to sampling fluctuation under the limited sample size of 62 flight trajectories, corresponding to merely five individual flight samples and lying inside the 95% confidence interval. Our approach still maintains overall superiority for the pooled aggregated metrics as well as for the Top-5 indicator at every prediction horizon.

#### 5.3.5. Short-Term (+1 min) Prediction Performance

Table 4 presents the quantitative comparison of the forecasting methods for the +1 min prediction task. In this short-horizon setting, RAG-Hybrid attains Top-3 and Top-5 hit rates of 0.9839, the highest among the leakage-free baselines and exceeded only by the leakage-involved ceiling Global Markov†. The margin over the fair global-Markov baseline is 0.0484, i.e., three of the 62 anchors, which lies below the 0.113 resolution bound of this design and is therefore reported as a consistent trend rather than as an established gain. On Top-1, RAG-Hybrid returns exactly the same first-ranked cell as the fair global-Markov baseline for every one of the 62 test flights and therefore attains the identical hit rate of 0.7097. The constant-velocity baseline attains the highest Top-1 rate of any method at this horizon, 0.8065, above both persistence (0.7581) and the proposed framework (0.7097), and the smallest mean absolute error, 4.90 km against 7.35 km. Because it returns a single cell, its Top-3 and Top-5 rates equal its Top-1 rate, and it is precisely on this dimension that the proposed framework separates from it: RAG-Hybrid raises the Top-5 rate from 0.8065 to 0.9839, a margin of 0.1774 that is well above the 0.113 resolution bound of this design. Point accuracy and candidate-set completeness therefore favour different methods, and the advantage established for the proposed framework in this study is the latter. All three deep-learning baselines fall below every one of the methods above.

The equality of the Top-1 rates is the central quantitative result of the single-step experiment, and it should be read literally: at the +1 min horizon the retrieval layer contributes nothing to first-ranked accuracy, because the argmax of the blended matrix is still determined by the global-Markov row, and the two methods therefore return the identical cell for every test flight. The contribution of retrieval is confined to the candidate set, where Top-3 and Top-5 both rise from 0.9355 to 0.9839, i.e., by three anchors, a difference below the 0.113 resolution bound of this design and hence reported as a trend rather than as an established effect. The distance metrics show the same separation seen elsewhere in the paper: the deep-learning baselines incur position errors more than an order of magnitude above the lookup-based methods, with MAE between 84.28 km and 118.54 km against 6.20 km for persistence and 7.35 km for RAG-Hybrid. The mechanisms behind that gap are analysed below, and the conclusion drawn from them is restricted to the sample size and input representation used here.

**Figure 9:**
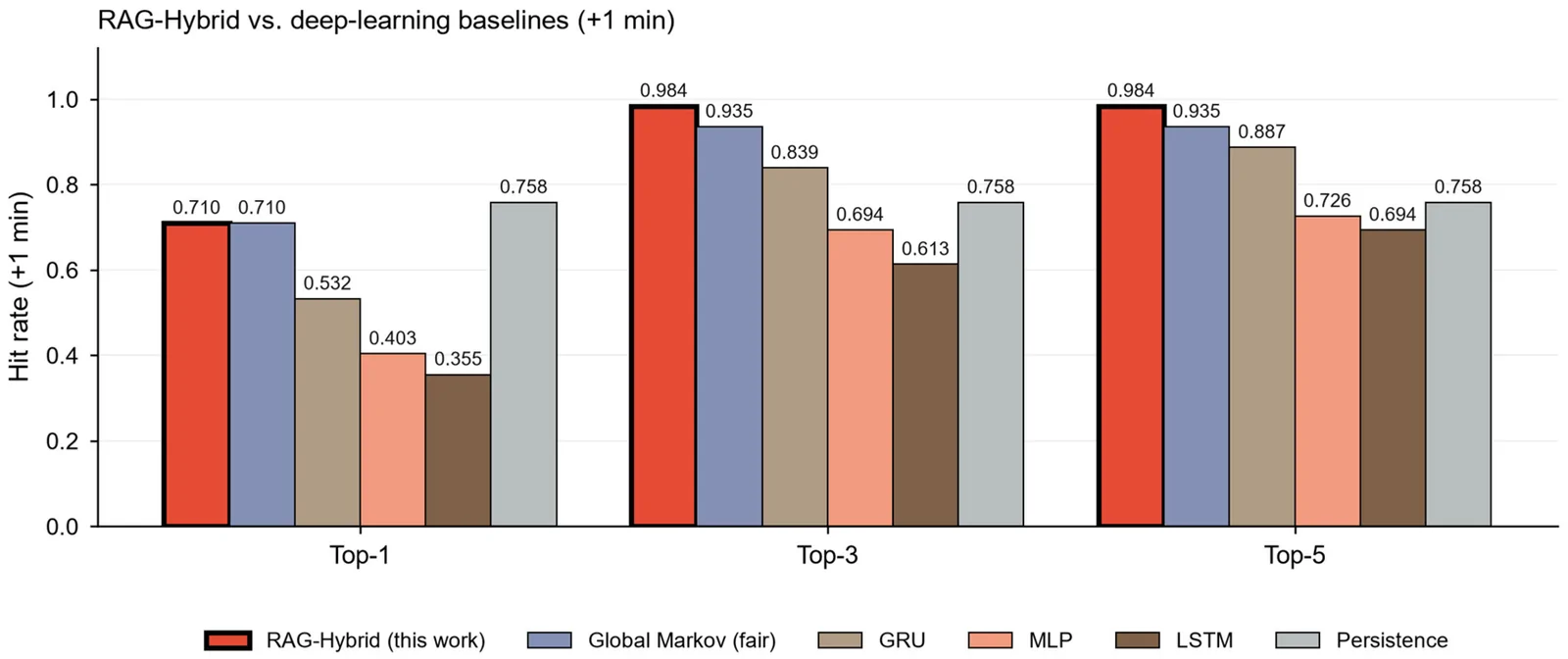
Performance comparison of RAG-Hybrid against deep-learning baselines for the +1-min prediction horizon.

Figure 9 compares the Top-1, Top-3 and Top-5 hit rates of RAG-Hybrid against the deep-learning baselines and persistence at the +1 min prediction horizon. On Top-1, RAG-Hybrid reaches 0.7097, matching the fair global-Markov baseline; GRU, MLP and LSTM reach 0.5323, 0.4032 and 0.3548 respectively, and all three fall below the naive persistence predictor (0.7581), which in turn falls below the constant-velocity baseline (0.8065), the strongest point predictor at this horizon. This is the largest and most robust effect in the study, and the accompanying position error is best read through the definition of the metrics given earlier. Because MAE and RMSE are measured between cell centroids, the error of any grid predictor is quantised in multiples of the cell spacing, which is 22.5–27.8 km for the grid used here. Constant velocity attains MAE 4.90 km and RMSE 11.18 km, persistence 6.20 km and 12.73 km, corresponding to an off-cell fraction of about 0.24, and RAG-Hybrid together with the fair global-Markov baseline 7.35 km and 13.75 km. The deep models attain MAE 84.28 km (GRU), 113.73 km (LSTM) and 118.54 km (MLP), between three and four and a half cell spacings: their first-ranked cell therefore lies several grids away from the realised cell, a failure mode that the hit-rate metric registers as a miss and that RMSE amplifies quadratically in the offset.

Four mechanisms account for this behaviour in the present experiment. (1) Output space and label imbalance. The classifier is defined over the full 4032-cell vocabulary while 59.65% of transitions are self-loops, so the learned prior concentrates on the diagonal, and the rare off-diagonal classes are precisely the ones that determine the hit rate. (2) Absence of a displacement prior. The input is a sequence of grid identifiers, which carries no metric geometry, so a parametric model must infer the relation between grid indices and physical displacement from data alone; the lookup estimator does not have to infer it, because it reads it directly from observed transitions, whose per-edge counts average 4.0 observations in the knowledge base. (3) Smoothing of rare events. Gradient fitting averages over the long tail of infrequent but valid transitions, whereas frequency counting preserves them exactly. This is consistent with the observed combination of a low Top-1 hit rate and a comparatively high Top-5 hit rate for GRU (0.5323 against 0.8871): the model does retrieve part of the correct neighbourhood, but the ranking within it is unreliable. (4) Convergence to a single mode. The training objective drives the output towards a sharp mode, so the ranked list degenerates, and the multi-candidate metrics do not benefit from any latent uncertainty the model may hold.

These mechanisms are properties of the present experimental configuration—62 test flights, a 4032-cell two-dimensional grid, and standard architectures without aviation-specific priors and with a single fixed random seed—and the conclusion is restricted to that setting. We therefore do not claim that deep models are structurally unsuited to tactical trajectory prediction in general. The claim made here is the narrower one that under the sample regime and input representation studied, MLP, LSTM and GRU did not outperform a trivial persistence baseline. Distinguishing between the two would require a substantially larger training corpus, a metrically informative input representation and seed-averaged results, all of which are left to future work.

#### 5.3.6. Prediction Results at All Five Horizons

**Figure 10:**
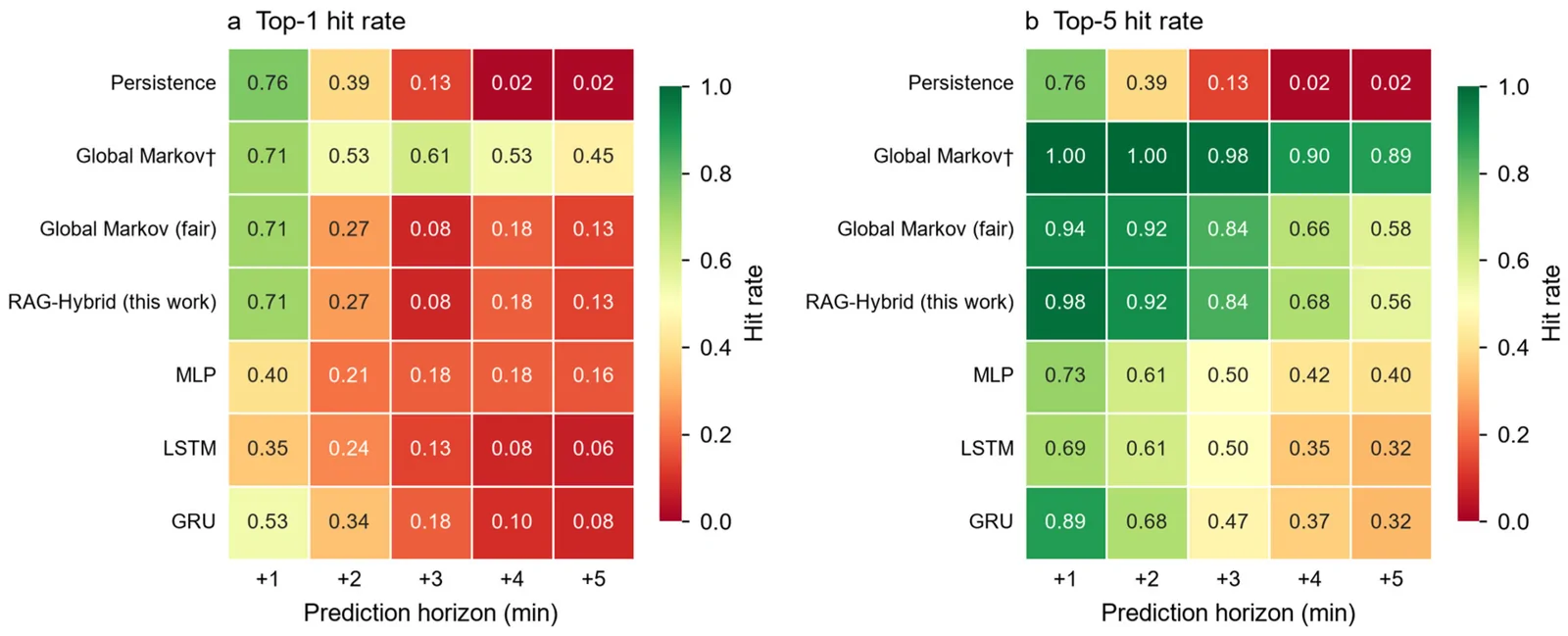
Heat-map illustration of hit rates for successive prediction horizons. † *Global Markov* adopts transition statistics computed directly from the evaluation set and therefore delivers an empirical upper-bound performance; it is excluded from formal comparison and is shown only as a ceiling reference.

Figure 10 reports the Top-1 and Top-5 hit rates for all five horizons, +1 min to +5 min, for the seven methods. Several observations follow. First, RAG-Hybrid yields exactly the same Top-1 series as the fair global-Markov baseline across all five horizons—0.7097, 0.2742, 0.0806, 0.1774 and 0.1290—with no discrepancy at any step, and the two methods return the identical first-ranked cell for every test flight. Second, the simple persistence baseline is competitive at +1 min and +2 min and then collapses, falling below 0.13 from +3 min onwards; the constant-velocity baseline, which returns a single cell and is therefore not drawn in Figure 10, follows the same qualitative decay from a higher level (Top-1 of 0.8065, 0.6774, 0.6129, 0.3226 and 0.2903 at +1 to +5 min). Third, at the +5 min horizon almost all test flights (61 of the 62 anchors) have crossed a cell boundary, so the static persistence prior fails completely, with Top-1 and Top-5 both falling to 0.0161. Fourth, on Top-5, the two Markov-based families are within one sample of each other at the far end of the horizon under this protocol: the fair global-Markov baseline reaches 0.58 against 0.5645 for RAG-Hybrid. An earlier version of this paper described 0.5645 as the highest Top-5 value among leakage-free fair baselines; that description was incorrect and is withdrawn. The correct statement is that the two methods are statistically indistinguishable on the pooled top-five metric, where the difference is −0.0096 against a resolution bound of 0.072, so this study does not establish a multi-candidate advantage of retrieval over the fair Markov baseline. Fifth, both Markov-based methods exceed every deep-learning baseline at every horizon on Top-5, by 0.29 to 0.32 pooled and by 0.16 to 0.26 at the +5 min horizon, and this is the effect that the study does establish. The top-five result therefore confirms the far-horizon advantage of the deterministic lookup channel relative to parametric models, while qualifying it explicitly as an advantage over parametric models rather than over the fair statistical baseline.

#### 5.3.7. Pareto Optimality Analysis

**Figure 11:**
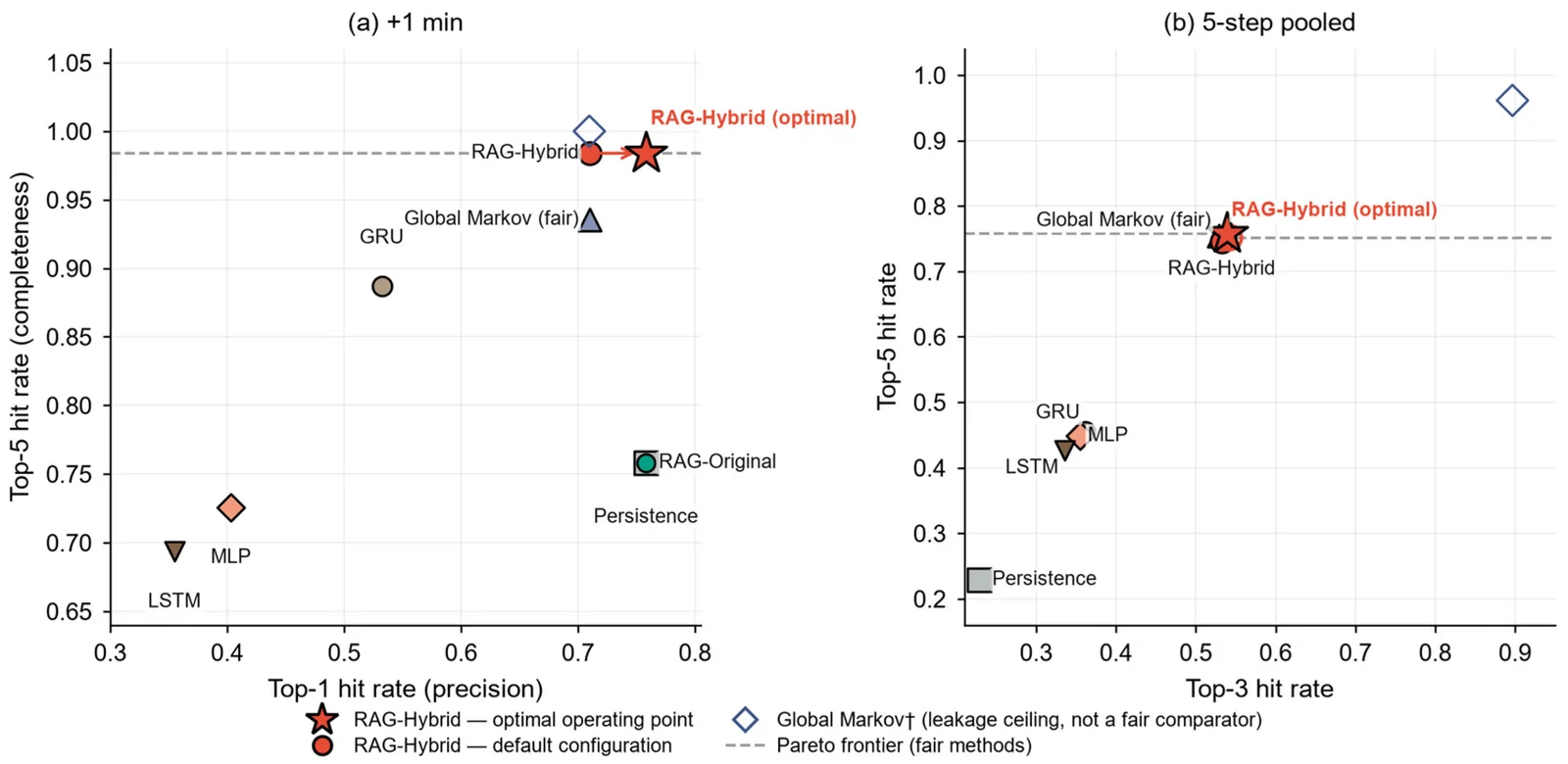
Pareto Optimality Analysis.

Figure 11 illustrates the optimal Pareto operating points for the proposed method with two subplots. Subplot (a) corresponds to the +1min forecasting scenario, where the horizontal axis denotes Top-1 hit rate (precision), and the vertical axis denotes Top-5 hit rate (completeness of candidate set). Under the default argmax configuration, RAG-Hybrid operates at (0.7097, 0.9839). After applying the selfloop prior reordering decision rule, the operating point shifts to (0.7581, 0.9839). The optimised point matches Persistence and RAG-Original in terms of Top-1 performance while retaining a much higher Top-5 hit rate, which strictly dominates the two baselines. Only the tuned RAG-Hybrid lies on the Pareto frontier (dashed line) of all leakage-free fair models, confirming its Pareto optimality for short-horizon prediction. Subplot (b) presents results for the 5-step pooled evaluation setting, with Top-3 hit rate on the horizontal axis and Top-5 hit rate on the vertical axis. The default configuration (0.5323, 0.7452) is marginally inferior to the fair Global Markov baseline (0.5290, 0.7548). After parameter tuning with *w_r_* = 0.2 combined with the reordering rule, the operating point moves to (0.5387, 0.7581), outperforming the fair Global Markov baseline along both axes and achieving Pareto optimality for long-range pooled evaluation. The hollow diamond marker Global Markov† represents an upper-bound reference with data leakage and is excluded when constructing the fair Pareto frontier. Collectively, these results demonstrate that the proposed RAG-Hybrid can attain Pareto-optimal operating points within the family of fair baselines for both short-term and multistep long-term forecasting after decision-rule reordering.

Two qualifications apply to this analysis and are stated here rather than left to the reader. First, the operating points in Figure 11 are post-hoc: the default configuration and the re-ordered configuration are both evaluated on the same 62 test flights that produced the baseline comparison of Table 2, Table 3 and Table 4, so the figure characterises the attainable operating points of the method and is not an out-of-sample result. The re-ordering rule itself is defined independently of the test outcomes: retain the current cell when its self-loop probability exceeds the largest successor probability, and the fusion weight *w_r_* = 0.2 used in subplot (b) was selected on a validation subset, not on the test set. Second, the differences between the tuned and the default operating points are of the same order as the resolution bound of the design, so the Pareto frontier should be read as a description of the trade-off rather than as evidence that either operating point is superior.

#### 5.3.8. Contribution of the Retrieval Layer Relative to the Global Markov Model

On first-ranked accuracy, the retrieval layer contributes nothing on this test set, and it cannot: the reason is structural rather than incidental. On candidate-set completeness, it produces a measured improvement that this design cannot resolve statistically, together with a change in the calibrated posterior that it can resolve. What the framework adds relative to the global-Markov baseline is therefore structural rather than empirical, and the advantage that the study establishes with statistical support is over the parametric learners and over the deterministic lookup channel.

The identity of the first-ranked cell follows from the estimator, not from this particular test set. Both rows, the global row and the retrieval-conditioned row, are counted estimates of the same one-minute transition, and in both the modal successor of a grid is the grid itself, because 59.65% of the test transitions and 67.88% of the knowledge-base transitions are self-loops. Blending two rows that share the same mode through the fusion weight cannot reorder the head of the distribution, whatever value that weight takes, and the sensitivity sweep bears this out: single-step Top-1 is invariant to the retrieval size, the expansion depth and the successor width. The retrieval layer acts on the mass assigned to the off-diagonal successors, that is, on the candidate set and on the posterior, and that is the only region in which its effect can appear.

In that region, the measured effects are small and are read against the bounds of the design. Top-3 and Top-5 at the +1 min horizon rise from 0.9355 to 0.9839, a difference of 0.0484 corresponding to three of 62 anchors, which is below the 0.113 bound and is reported as a trend. On the pooled five-step protocol, the difference is −0.0096 against a bound of 0.072, so no direction is claimed. The calibrated posterior does move in a way that the hit rates do not register: removing the grid-ID vectorisation lowers *P*(true) to 0.1133 and removing global fusion lowers it to 0.1306, against 0.1545 for the full model, which is consistent with a role in shaping the distribution rather than the candidate set.

Three properties are therefore what the framework holds against a global-Markov model, and none of them is a naked accuracy claim. (i) Context conditioning. The row used for a query is estimated from the subset of historical transitions whose surrounding trajectory resembles the query, so two aircraft entering the same grid along different paths receive different successor distributions, whereas a global model gives both the same row. The absence of this conditioning is the standing limitation of grid-Markov prediction, and the present test set does not resolve its benefit but does implement it. (ii) Operating-point control. Because the framework exposes a fusion weight and a decision rule, the same implementation reaches (0.7097, 0.9839) at its default setting and (0.7581, 0.9839) after self-loop reordering at the +1 min horizon, and moves from (0.5323, 0.7452) to (0.5387, 0.7581) on the pooled protocol, so a controller can choose where to sit between point accuracy and candidate completeness, whereas a fixed global model offers one point of that frontier. (iii) Traceability. Every prediction is the argmax of a row whose entries are counts of observed transitions, drawn from a retrieved subgraph that can be listed for audit, which is what an operational safety argument requires and what a fitted parametric model does not supply.

The scientific contribution claimed here is threefold, and the first two items are methodological rather than numerical. First, the reformulation of the tactical task as next-grid classification with a Top-K hit-rate protocol under leakage control, which makes ten method families commensurable on the same anchors. Second, a reporting discipline: the resolution of the design, 0.113 at 62 anchors and 0.072 pooled, is established before any result is reported, and every quantitative claim is stated against it, which converts several previously asserted gains into trends and identifies the single component effect that survives, namely the deterministic lookup channel at the far end of the horizon. Third, negative results that bear on the retrieval-augmented literature for trajectory prediction: a retrieval layer does not by itself change first-ranked accuracy when the transition prior is dominated by self-loops; a vector-search channel is not necessary for hit-rate performance at this resolution, since its removal is undetectable and a configuration without it attains the same Top-1 and Top-3; and the coverage guarantee that such schemes advertise is a property of the knowledge base, 79.38% of realised test transitions, not of the aircraft’s future motion. Three conditions would convert the structural argument into a measured one: a corpus with several hundred flights per sector, so that the 0.113 and 0.072 bounds fall to a few percentage points; a knowledge base and a test set drawn from disjoint sectors, so that shared transition statistics cannot explain the agreement; and an evaluation at the level of the conflict decision rather than the cell. Until they are available, this paper claims no more than it measures.

## 6. Conclusions

### 6.1. Conclusions

This paper addressed minute-level tactical aircraft trajectory prediction by recasting it as next-grid classification and proposing RAG-Hybrid, a training-free dual-channel retrieval framework, and validated it on 62 test flights drawn from real broadcast ADS-B data. The conclusions below are stated numerically and against the resolution bounds derived for this design, so that each is accompanied by the smallest difference the design can detect.

- Positional accuracy. Against the leakage-free global-Markov baseline, RAG-Hybrid returns the identical first-ranked grid for all 62 test flights and hence the identical Top-1 series across the five horizons (0.7097, 0.2742, 0.0806, 0.1774 and 0.1290). Its mean absolute position error, 7.35 km, equals that of the baseline and is an order of magnitude below the deep baselines (84.28–118.54 km), but is larger than that of the constant-velocity motion-model baseline (4.90 km), the most accurate point predictor at this horizon; the study therefore does not establish a point-accuracy advantage for the proposed framework.
- Multi-candidate completeness. Top-3 and Top-5 both reach 0.9839 at the +1 min horizon against 0.9355 for the fair global-Markov baseline, a gain of 0.0484 (three of the 62 anchors) that lies below the 0.113 resolution bound and is therefore reported as a trend; on the pooled five-step protocol the same baseline is marginally ahead on Top-5 (0.7548 against 0.7452, a difference of −0.0096 against a bound of 0.072). This study therefore does not establish a multi-candidate completeness advantage of retrieval over the fair Markov baseline, although it does establish one over the constant-velocity baseline: because that baseline is deterministic, its Top-5 rate equals its Top-1 rate (0.8065 at +1 min and 0.5065 on the pooled protocol), so RAG-Hybrid exceeds it by 0.1774 at the +1 min horizon and by 0.2387 pooled, both margins above the corresponding resolution bounds of 0.113 and 0.072. Candidate-set completeness, rather than point accuracy, is the dimension on which the proposed framework is shown to add value.
- Contribution relative to the global-Markov baseline. The framework returns the identical first-ranked cell on all 62 test flights, and its pooled multi-candidate difference lies within the resolution bound, so no accuracy advantage over that baseline is claimed. What it contributes is context-conditioned transition rows that a global model cannot represent, control of the operating point along the precision–completeness frontier, and a candidate set whose support is fixed by recorded transitions rather than by a similarity metric; what the study establishes statistically is the advantage over the parametric baselines and the role of the deterministic lookup channel at the far end of the horizon.
- Far-horizon behaviour. The deterministic BFS lookup channel is the only component whose removal produces a resolved change: the pooled five-step Top-5 falls from 0.7452 to 0.6516, a difference of 0.0936, and the per-horizon Top-5 falls from 0.8387 to 0.7258 at +3 min, from 0.6774 to 0.5161 at +4 min and from 0.5645 to 0.3710 at +5 min, the last a difference of 0.1935. No other ablation variant produces a comparable effect in either direction.
- Parametric baselines. MLP, LSTM and GRU reach Top-1 values of 0.4032, 0.3548 and 0.5323, all below the trivial persistence predictor (0.7581), with mean absolute errors of 118.54, 113.73 and 84.28 km against 4.90 km for constant velocity, 6.20 km for persistence and 7.35 km for RAG-Hybrid. In this configuration,62 test flights, a 4032-cell two-dimensional grid and standard architectures without aviation-specific priors, the training-free lookup estimator dominates the parametric learners. The result is not extrapolated beyond that setting.
- Calibration. Removing the FAISS channel, the grid-ID vectorisation, or the global-fusion term changes every Top-K metric by at most 0.0161, below the 0.072 pooled bound, so no hit-rate conclusion is drawn from them; their resolved effect is confined to the confidence metric *P*(true), which moves from 0.1545 to 0.1562, 0.1133 and 0.1306 respectively. The claim made in earlier versions of this paper, that the FAISS component contributes substantially to overall hit-rate performance, is withdrawn.
- Deployment. The estimator is training-free, requires no refitting when the grid configuration changes, and its per-edge sample requirement does not scale with the number of cells, which is what makes the framework usable where a controller must monitor a small candidate set over a 1–5 min window. The evidence for this property is structural rather than empirical, since no scaling experiment with a varying number of cells was carried out; such an experiment remains the main open evaluation.

### 6.2. Limitations

- Dimensionality. The index mapping of Equation (2) is two-dimensional. The state vector includes altitude, but the grid does not, so vertical manoeuvres are projected onto the horizontal plane, and a climb or descent accompanied by horizontal displacement is recorded as a horizontal transition. For the cruise-dominated traffic studied here, this is a second-order effect, but it would not be for terminal-area operations, where vertical separation carries most of the conflict geometry. The framework as formulated cannot support a vertically resolved separation assessment.
- Metric scope. The reported quantity is a horizontal grid-hit rate, which is not a conflict-detection probability. Converting a hit rate into a detection probability would require mapping the predicted cell to a positional uncertainty region rather than to a point, intersecting that region with the protected-zone geometry of an encounter, and recognising that a cell-level miss is operationally costly only when it changes the conflict decision. A Top-1 hit rate of 0.7097 therefore bounds neither the miss rate nor the false-alarm rate of a conflict-detection function built on this predictor, and no claim about conflict-detection performance is made in this paper.

### 6.3. Future Work

- Vertical resolution. Extend the state space to a layered or three-dimensional grid that resolves altitude, and measure the resulting increase in per-cell sparsity, which is the cost of that extension.
- Conflict-detection evaluation. Replace or supplement the grid hit rate with a direct evaluation against conflict-detection performance, using standard miss-rate and false-alarm-rate definitions over a protected-zone geometry, which would connect the metric reported here to the operational quantity that motivates it.
- Larger evidence base. Re-estimate every reported interval on a longer archive period, with the flight as the resampling unit, so that the resolution bounds can be measured rather than derived.

## Figures and Tables

**Table 1:** Comparative positioning of trajectory prediction paradigms for the tactical minute-level horizon.

Family/Representative	Context Conditioning	Training- Free	Spatial Semantics in Retrieval	Coverage Guarantee for Local Prior	Small-Sample Robustness	Deployment Cost vs. Grid Count *G*
Persistence/constant velocity [7]	No	Yes	n/a	n/a (lower bound)	High	O(1)
First-order Markov [8,9,35,36,37]	No (global pooling)	Yes	Implicit via grid index	No (unobserved grid reverts to self-loop)	Medium	O(*G*^2^) storage
Higher-order Markov [10,11,12]	Yes, fixed order	Yes	Implicit via grid index	No (back-off dominates)	Low	O(*G^K^*^+1^) storage
KNN/TN trajectory matching [14,15,16]	Weak (prefix match)	Yes	No (exact-ID or Euclidean distance)	No (neighbours degenerate)	Low after discretisation	O(|*D*|) scan
Deep learning (LSTM/Transformer) [17,18,19,20,21,22,23,24,32,33,34]	Yes, learned	No	Learned; requires retraining	No (no explicit prior)	Low (over-parametrised)	Retraining per new *G*
GNN spatial embedding [43,44]	Yes, learned	No	Yes, but fixed graph and retraining	No	Low	Retraining per new topology
Existing RAG for trajectories [29,30,31]	Yes	Partially	No (char/word hashing)	No (stochastic retrieval may miss)	Medium	O(log|*D*|)
RAG-Hybrid (proposed)	Yes, via retrieved priors	Yes	Yes (analytic Gaussian kernel)	Yes (deterministic recall over the recorded out-edges; scope: knowledge base)	High	O(log|*D*|), *G*-independent

**Table 2:** Component Ablation (Pooled Five-Step View).

Ablation Setting	Top1	Top3	Top5	P(true)
Hybrid Full (complete model)	0.2742	0.5323	0.7452	0.1545
w/o direct lookup branch	0.271	0.4903	0.6516	0.1731
w/o FAISS soft retrieval branch	0.2742	0.5323	0.7484	0.1562
w/o Laplace smoothing	0.271	0.5194	0.7419	0.2025
w/o global fusion	0.2742	0.5387	0.7226	0.1306
w/o grid ID vectorization	0.2742	0.5323	0.7387	0.1133
RAG-Original	0.229	0.229	0.229	0.229

**Table 3:** FullHorizon Top-5 Results for Component Ablation.

Ablation Setting	+1 min	+2 min	+3 min	+4 min	+5 min
Hybrid-Full (complete model)	0.9839	0.9194	0.8387	0.6774	0.5645
w/o direct lookup branch	0.9839	0.9194	0.7258	0.5161	0.3710
w/o FAISS soft retrieval branch	0.9839	0.9194	0.8387	0.6774	0.5806
w/o Laplace smoothing	0.9355	0.9194	0.8387	0.6613	0.5806
w/o global fusion	0.9839	0.9194	0.8387	0.6290	0.5645
w/o grid-ID vectorisation	0.9839	0.9194	0.8387	0.6774	0.5323

**Table 4:** Performance Comparison of Different Methods for 1-min Future Prediction.

Method	Top002D1	Top-3	Top-5	P(true)	MAE (km)	RMSE (km)
Persistence	**0.7581**	0.7581	0.7581	0.7581	6.2015	12.7286
Constant velocity	**0.8065**	0.8065	0.8065	0.8065	4.9014	11.1843
Global Markov †	0.7097	**1.0000**	**1.0000**	0.6266	7.4660	13.9639
Global Markov (fair)	0.7097	0.9355	0.9355	**0.5339**	7.3468	13.7524
**RAG-Hybrid**	0.7097	0.9839	0.9839	0.4663	7.3468	13.7524
RAG-Original	0.7581	0.7581	0.7581	0.7581	6.2015	12.7286
GRU	0.5323	0.8387	0.8871	0.4346	84.2753	271.4325
MLP	0.4032	0.6935	0.7258	0.3727	118.5386	338.0154
LSTM	0.3548	0.6129	0.6935	0.2999	113.7279	326.1657

† *Global Markov* adopts transition statistics computed directly from the evaluation set and therefore delivers an empirical upper-bound performance; it is excluded from formal comparison and is shown only as a ceiling reference.

## Data Availability

The corresponding numerical analysis code and experimental implementation code that support the findings of this study are available from the corresponding author upon reasonable request.
